# The ion channel TRPM7 regulates zinc-depletion-induced MDMX degradation

**DOI:** 10.1016/j.jbc.2021.101292

**Published:** 2021-10-08

**Authors:** Herui Wang, Bin Li, Kulsum Asha, Ryan L. Pangilinan, Asha Thuraisamy, Harman Chopra, Susumu Rokudai, Yong Yu, Carol L. Prives, Yan Zhu

**Affiliations:** 1 Department of Biological Sciences, St John's University, Queens, New York, USA; 2 Department of Molecular Pharmacology and Oncology, Gunma University, Gunma, Japan; 3 Department of Biological Sciences, Columbia University, New York, New York, USA

**Keywords:** MDMX, TRPM7, zinc depletion, 20S proteasome, breast cancer, CZM, capzimin dimer, MEF, mouse embryo fibroblast, TRPM7, transient receptor potential melastatin 7

## Abstract

Zinc deficiency has been linked to human diseases, including cancer. MDMX, a crucial zinc-containing negative regulator of p53, has been found to be amplified or overexpressed in various cancers and implicated in the cancer initiation and progression. We report here that zinc depletion by the ion chelator TPEN or Chelex resin results in MDMX protein degradation in a ubiquitination-independent and 20S proteasome-dependent manner. Restoration of zinc led to recovery of cellular levels of MDMX. Further, TPEN treatment inhibits growth of the MCF-7 breast cancer cell line, which is partially rescued by overexpression of MDMX. Moreover, in a mass-spectrometry-based proteomics analysis, we identified TRPM7, a zinc-permeable ion channel, as a novel MDMX-interacting protein. TRPM7 stabilizes and induces the appearance of faster migrating species of MDMX on SDS-PAGE. Depletion of TRPM7 attenuates, while TRPM7 overexpression facilitates, the recovery of MDMX levels upon adding back zinc to TPEN-treated cells. Importantly, we found that TRPM7 inhibition, like TPEN treatment, decreases breast cancer cell MCF-7 proliferation and migration. The inhibitory effect on cell migration upon TRPM7 inhibition is also partially rescued by overexpression of MDMX. Together, our data indicate that TRPM7 regulates cellular levels of MDMX in part by modulating the intracellular Zn^2+^ concentration to promote tumorigenesis.

Zinc is the second most abundant transition metal in living organisms (1). It plays a crucial role in many biochemical processes through both structural and catalytic functions. It has also been shown to have a direct regulatory role in cellular signaling pathways (2). Zinc deficiency is linked to a variety of human cancers such as bladder, breast, esophageal, head and neck, prostate, and skin cancer (3).

p53, a zinc-containing transcription factor, is the most frequently mutated tumor suppressor protein in human cancer (4). The role of zinc in stabilizing wild-type p53 structure and loss of bound zinc in cancer-derived mutant p53 proteins has been well documented (5, 6, 7, 8). Two crucial negative regulators of p53, the homologous proteins, MDM2 and MDMX, are also zinc-containing proteins. Each has a C4 zinc-finger domain in their central region and a RING-type zinc finger domain at their C-terminus (9). The C-terminal RING domain of MDM2 but not MDMX possesses an intrinsic E3 ubiquitin ligase activity and MDMX regulates MDM2 E3 ligase activity through RING-RING interactions (10). Through the RING-containing E3 ligase activity, MDM2 promotes ubiquitination and 26S proteasome-mediated degradation of both p53 and MDMX in addition to several other cellular proteins (11). Zinc chelation or RING finger mutations abolish the E3 ligase activity of MDM2 (12). Moreover, it has been reported that MDM2 facilitates p21 (13, 14) or Rb (15) degradation through its binding to the c8 alpha subunit of the 20S proteasome independent of its E3 ligase function. The central region of MDM2 contains a C4 zinc-finger domain that has been shown to interact with multiple MDM2 regulatory proteins (16, 17). Several cancer-associated mutations have been identified in the MDM2 C4 zinc-finger domain to disrupt the MDM2-ribosomal protein interaction and attenuate MDM2-mediated p53 degradation (18). The MDMX RING domain can repress the proliferation of p53-null cells, while the zinc-finger domain of MDMX suppresses genome instability and tumor growth (19).

The TRPM7 (transient receptor potential melastatin 7) is a bifunctional protein with both ion channel and α-type protein kinase feature (20). TRPM7 is required for early embryonic development (21, 22) and is abnormally overexpressed in various cancer cells (23). As a cation channel, TRPM7 conducts physiologically essential metal cations such as Zn^2+^, Mg^2+^, and Ca^2+^ as well as environmentally toxic metals such as Ni^2+^, Cd^2+^, and Ba^2+^ with particularly high permeation of Zn^2+^ (24). The C-terminal kinase domain of TRPM7 can be cleaved and translocated to the nucleus, where it can bind multiple components of chromatin remodeling complexes as well as several transcription factors with zinc-binding domains (25, 26). It has been hypothesized that TRPM7-mediated modulation of intracellular Zn^2+^ concentration leads to epigenetic chromatin covalent modifications that affect gene expression patterns (25, 27).

Here, we report that zinc depletion results in MDMX degradation in a ubiquitination-independent and 20S proteasome-dependent manner. Adding back zinc recovers the cellular levels of MDMX while depletion of TRPM7 attenuates the effects of zinc. In addition, we show that TRPM7 interacts with and induces changed migration of the MDMX polypeptide upon denaturing polyacrylamide gel electrophoresis (SDS-PAGE) that requires the TRPM7 channel function. Moreover, TRPM7 inhibition decreases MCF-7 cancer cell growth and migration, while MCF-7 cells with MDMX overexpression are more resistant to TRPM7 inhibition-mediated cell migration suppression. Together, our results indicate that TRPM7 regulates cellular levels of MDMX in part by modulating intracellular zinc concentration, which in turn may provide a new therapeutic target for combinational cancer treatment.

## Results

### Zinc depletion by TPEN reduces cellular levels of MDMX

**Figure 1:**
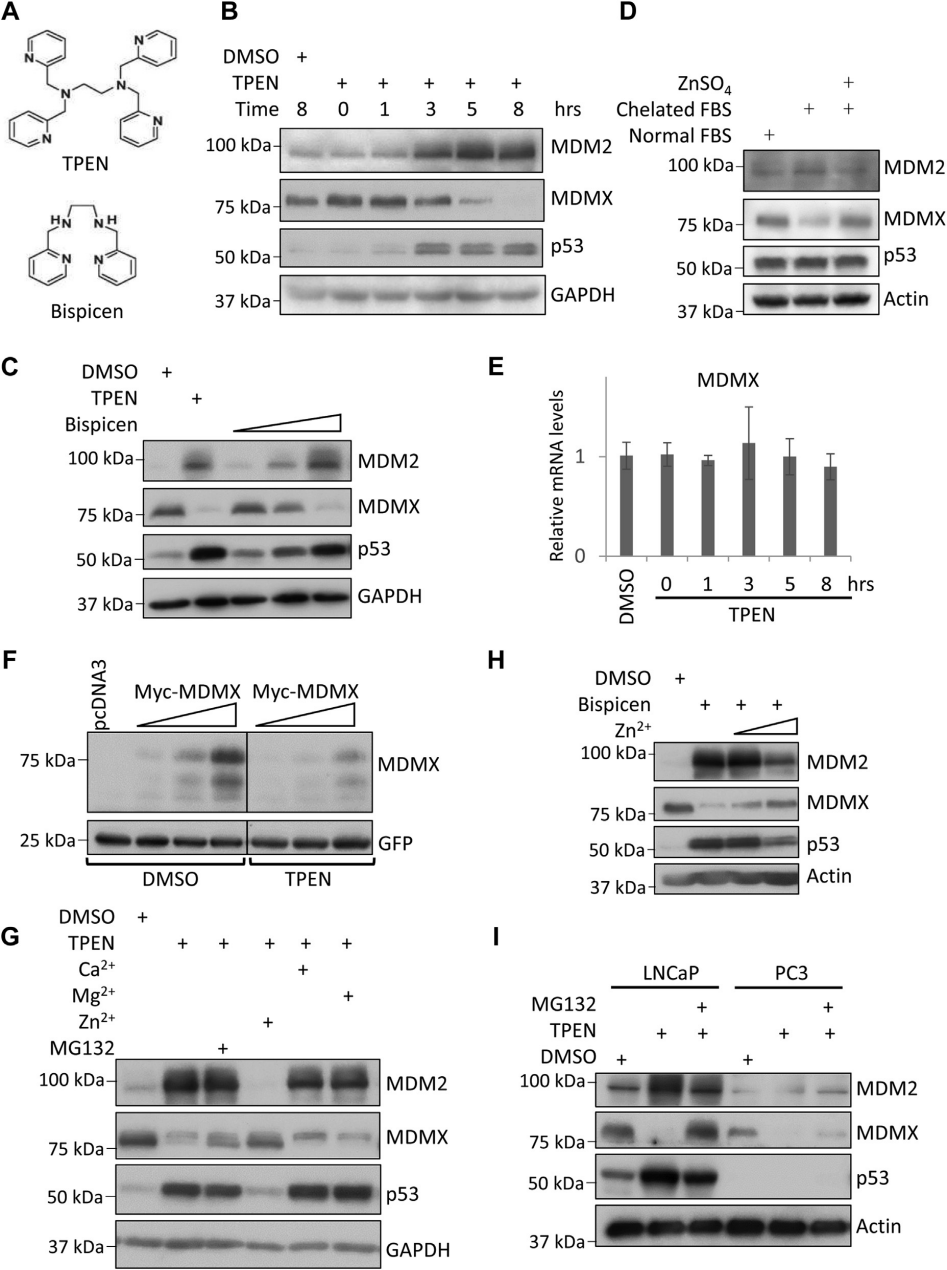
**Zinc depletion represses MDMX at protein level.***A*, structural formulas of zinc chelators TPEN and Bispicen. *B*, TPEN reduces cellular levels of MDMX but increases MDM2 and p53. MCF-7 cells were treated with DMSO (control) or 5 μM TPEN and harvested at indicated time points. *C*, bispicen reduces cellular levels of MDMX. MCF-7 cells were treated with 5 μM TPEN or increasing dosages of Bispicen (10, 50, or 100 μM) for 8 h. *D*, zinc depletion by Chelex resin reduces cellular levels of MDMX. MCF-7 cells were incubated in DMEM with 10% of normal FBS, DMEM with 10% of Chelex resin-treated FBS, or DMEM with 10% of Chelex resin-treated FBS supplemented with 5 μM ZnSO_4_ for 2 days. *E*, mRNA levels of MDMX remain constant after TPEN treatment. MCF-7 cells were treated and harvested as in panel *A*. Total RNA was extracted. Quantitative RT-PCR was carried out to check mRNA levels of MDMX. No statistical significance was observed. *F*, TPEN reduces levels of ectopically expressed MDMX. HEK293 T cells were transfected with increasing amounts of Myc-MDMX plasmids (0.15, 0.25, or 0.5 μg). GFP plasmids (0.05 μg) were also included as transfection control. Twenty-four hours after transfection, the transfected cells were treated with 5 μM TPEN for 8 h. *G*, adding back Zn^2+^ but not Ca^2+^, Mg^2+^ reverses the effect of TPEN on the cellular levels of MDMX, MDM2, and p53. MCF-7 cells were treated with 5 μM of TPEN for 5 h and then 25 μM CaCl_2_, MgCl_2_, ZnSO_4_, or MG-132 was added and incubated for 5 h. *H*, adding back Zn^2+^ reverses the effect of bispicen on cellular levels of MDMX, MDM2, and p53. MCF-7 cells were treated with 100 μM bispicen for 5 h then 5 or 25 μM ZnSO4 was added. Five hours later, the cells were harvested and total cell lysates were analyzed by immunoblotting with indicated antibodies. *I*, TPEN reduces cellular levels of MDMX in prostate cancer cells. LNCaP and PC3 cells were treated with 5 μM TPEN alone or in combination with 25 μM MG132 for 5 h. The cells were harvested and total cell lysates were analyzed by immunoblotting with indicated antibodies. All immunoblotting experiments were repeated at least twice to ensure reproducibility of the results.

In order to investigate the effects of zinc on the p53 signaling pathway, MCF-7 breast cancer cells were treated with 5 μM TPEN, an intracellular membrane-permeable zinc chelator (structure shown in Fig. 1*A*). We found that TPEN treatment induces p53 as previously reported (28). Intriguingly, the cellular levels of MDMX were dramatically reduced while the levels of MDM2 were simultaneously increased (Fig. 1*B*). Similarly, when we treated MCF-7 cells with bispicen, another zinc chelator (structure shown in Fig. 1*A*), we observed induction of p53 and MDM2 levels but reduction of MDMX levels (Fig. 1*C*). MDMX reduction was also observed in cells grown in zinc-deficient DMEM medium in comparison to the cells that were grown in zinc-deficient DMEM supplemented with 5 μM ZnSO_4_ or regular DMEM medium (Fig. 1*D*). Note that under this condition, no p53 or MDM2 induction was observed. This may be due to the fact that TPEN and bispicen are membrane-permeable agents, which can deplete intracellular zinc more efficiently than the extracellular zinc-deficient medium (29). TPEN and bispicen may also activate p53 through other mechanism(s). Nevertheless, our results demonstrate that cellular levels of MDMX are sensitive to zinc depletion. The reduction of MDMX levels most likely happens posttranscriptionally. Quantitative-RT-PCR analysis revealed that mRNA levels of MDMX remained fairly constant during the time period of TPEN treatment (Fig. 1*E*). Furthermore, we found that the levels of ectopically expressed MDMX proteins in HEK293T cells also decrease upon TPEN treatment (Fig. 1*F*).

To show that the effects of TPEN on cellular levels of MDM2, MDMX, and p53 are due to zinc depletion but not other divalent cations, we pretreated MCF-7 cells with 5 μM TPEN and added back 25 μM ZnSO_4_, CaCl_2_, or MgCl_2_. As shown in Figure 1*G*, adding back Zn^2+^ but not Ca^2+^ or Mg^2+^ reversed the effect of TPEN, as MDMX degradation was attenuated and the levels of p53 and MDM2 were decreased and went back to the same levels as in untreated cells. Similarly, adding back Zn^2+^ also reversed the effects of bispicen on cellular levels of MDMX, MDM2, and p53 (Fig. 1*H*).

The effect of TPEN was not limited to MCF-7 cells. As shown in Figure 1*I*, TPEN treatment led to reduced cellular levels of MDMX but increased levels of MDM2 and p53 in LNCaP prostate cancer cells. In PC3 cells, another p53-null prostate cancer cell line, we also observed TPEN-induced MDMX degradation. Together, our data demonstrate that zinc depletion has distinct effects on the three key components of the p53 signaling pathway. Most importantly, our results show that zinc depletion leads to MDMX degradation.

### TPEN-induced MDMX degradation is mediated by proteasome

**Figure 2:**
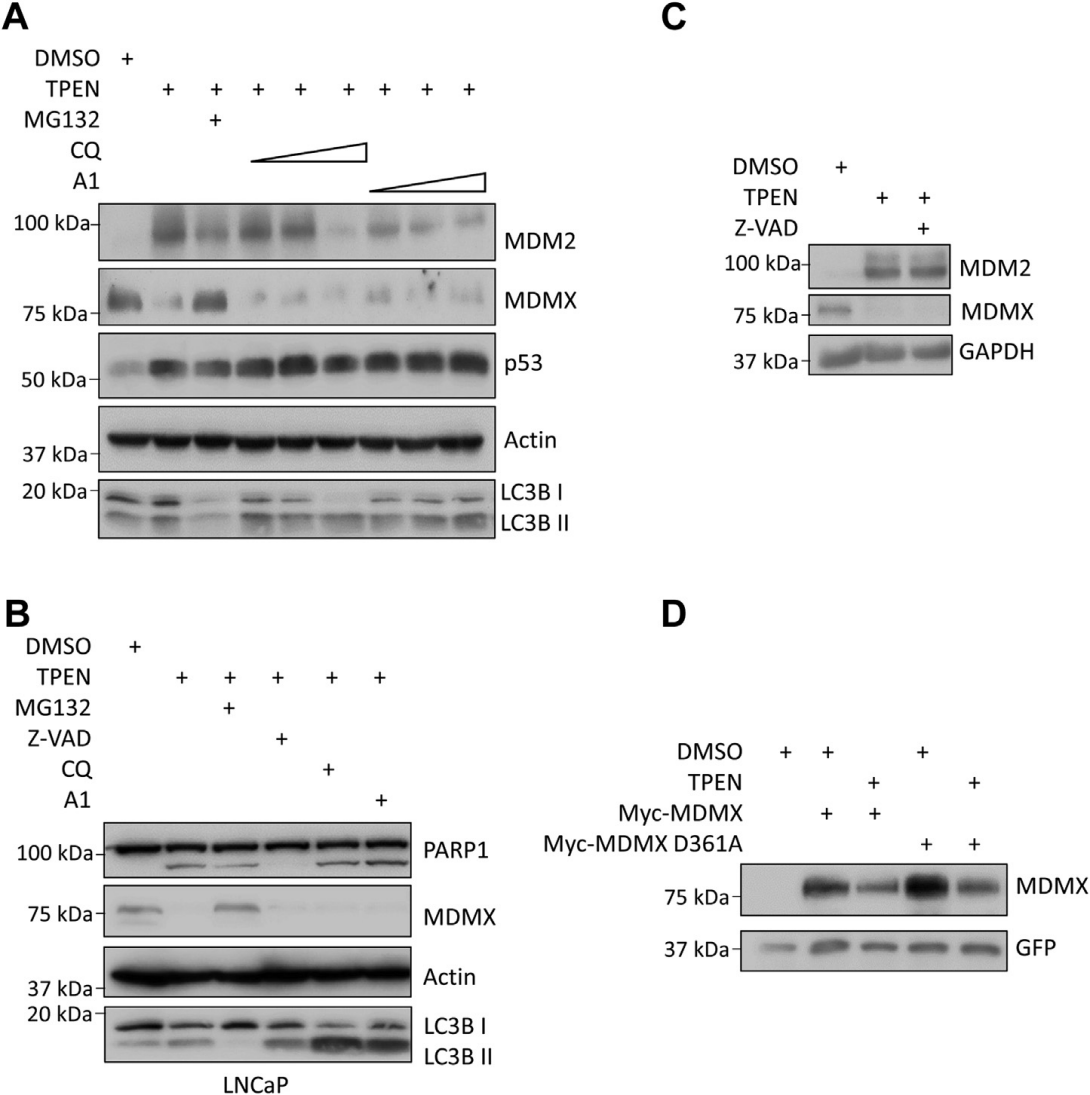
**TPEN-induced MDMX degradation is mediated by proteasome but not lysosome or caspase cleavage.***A* and *B*, proteasome inhibitor MG-132, but not lysosome inhibitors, protects MDMX from degradation. MCF-7 cells (*A*) were treated with DMSO (control) or 5 μM TPEN for 3 h and then MG-132 (25 μM), chloroquine (CQ: 25, 50, or 100 μM) or bafilomycin A1(A1; 50, 100, or 500 nM) was added. LNCaP cells (*B*) were treated with DMSO or TPEN (5 μM) for 3 h, then MG-132 (25 μM), Z-VAD-FMK (25 μM), chloroquine (CQ: 50 μM), or bafilomycin A1(A1; 100 nM) was added. Five hours later, the cells were harvested and total cell lysates were analyzed by immunoblotting with indicated antibodies. *C*, caspase inhibitor does not prevent MDMX from TPEN-induced degradation. MCF-7 cells were treated with 5 μM TPEN in the presence or absence of Z-VAD-FMK (25 μM) for 8 h (*D*) TPEN reduces levels of ectopically expressed MDMX caspase cleavage-resistant variant D361A. HEK293T cells were transfected with wild-type Myc-MDMX or Myc-MDMX D361A (0.15 μg). Twenty-four hours after transfection, transfected cells were treated with 5 μM TPEN for 8 h. All the immunoblotting experiments were repeated at least twice to ensure reproducibility of the results.

TPEN-mediated zinc chelation can cause oxidative DNA damage and chromosome breaks (30) and activate cytosolic caspase activity (31). It has been shown that proteasome-mediated degradation of MDMX occurs after DNA damage or ribosomal stress (32). In addition, it was reported that MDMX can be subjected to caspase cleavage at Glu361, leading to reduced cellular MDMX levels (33). Moreover, zinc chelation may lead to MDMX misfolding for lysosomal degradation. To examine the mechanisms that account for TPEN-induced MDMX reduction, we pretreated MCF-7 cells with 5 μM TPEN and then cotreated cells with the proteasome inhibitor MG-132 or two types of lysosome inhibitor chloroquine and bafilomycin A1. We found that only MG-132 but not chloroquine or bafilomycin A1 reversed the effect of TPEN on MDMX, suggesting that TPEN-induced MDMX degradation is mediated by the proteasome but not by lysosomal-mediated turnover (Fig. 2*A*). Similarly, in LNCaP cells, MG-132 but not chloroquine or bafilomycin A1 cotreatment reversed the effect of TPEN on cellular levels of MDMX (Fig. 2*B*). Note that both chloroquine and bafilomycin A1 inhibited LC3B-II degradation (Fig. 2, *A* and *B*), indicating their effectiveness under the experimental condition.

To test if caspase cleavage accounts for TPEN-mediated MDMX reduction, MCF-7 cells were treated with 5 μM TPEN in the presence or absence of the caspase inhibitor Z-VAD-FMK. We found that TPEN treatment led to reduced levels of MDMX regardless of the presence of Z-VAD-FMK (Fig. 2*C*). Similarly, Z-VAD-FMK had no effect on TPEN-induced MDMX reduction but inhibited TPEN-induced PARP cleavage in LNCaP cells (Fig. 2*B*). Additionally, we observed that TPEN treatment reduces the cellular levels of ectopically expressed wild-type MDMX as well as the mutant MDMX (D361A), a mutant that is resistant to caspase cleavage (Fig. 2*D*). Note that we detected a slight increase in ROS (Reactive Oxygen Species) levels in TPEN-treated cells in comparison to the DMSO-treated cells, but to a much lesser extent when compared with cells treated with pyocyanin, a cell-permeable compound capable of redox cycling (Fig. S1). Therefore, under our experimental conditions, caspase cleavage most likely does not contribute to TPEN-induced MDMX reduction.

### TPEN-induced MDMX degradation is mediated by the 20S proteasome and is independent of ubiquitination and MDM2

**Figure 3:**
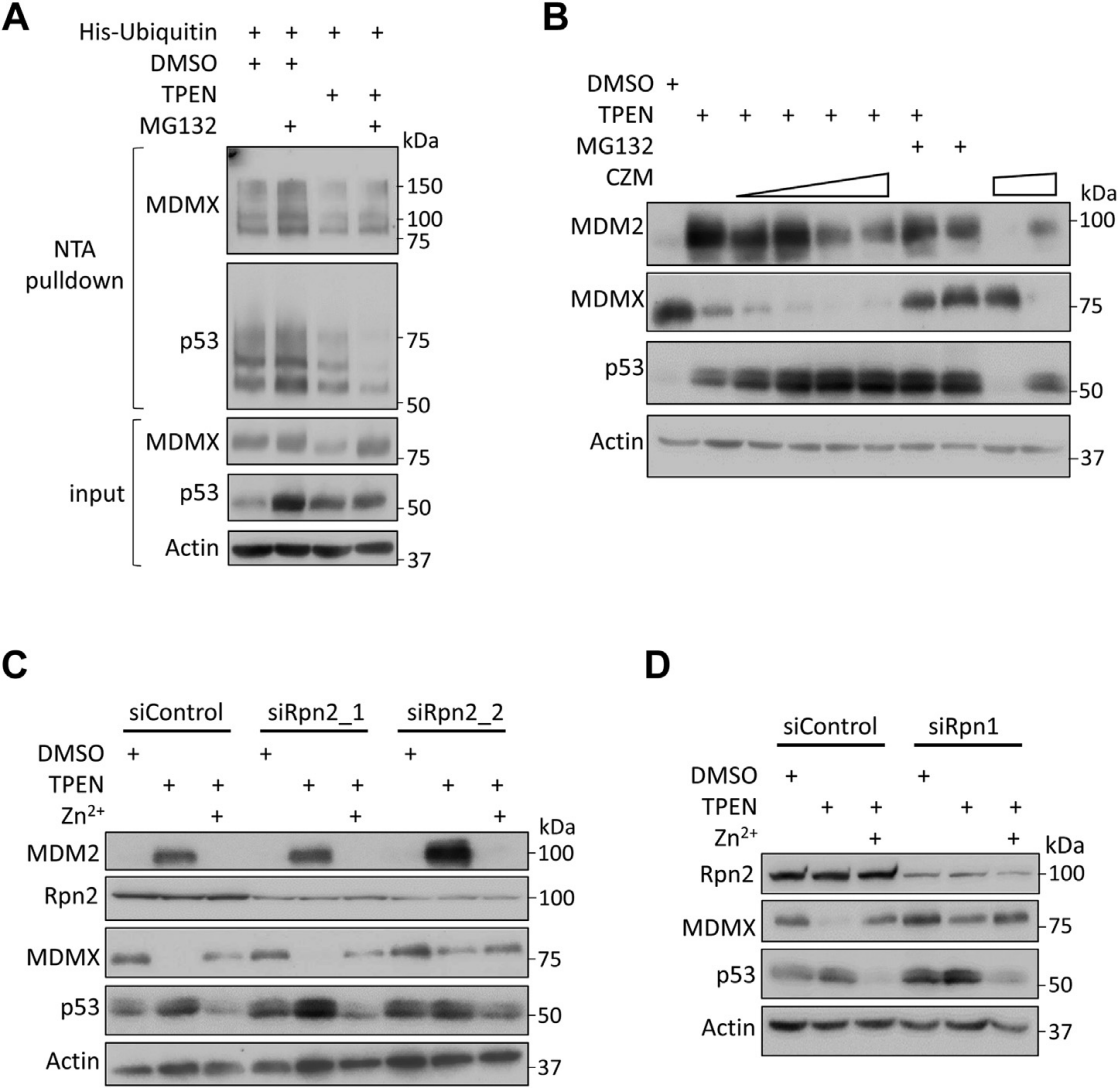
**TPEN-induced MDMX degradation is mediated by 20S proteasome, independent of ubiquitination.***A*, TPEN does not induce ubiquitination of MDMX. MCF-7 cells were transfected with His-ubiquitin (5 μg) plasmids. Twenty-four hours later, cells were treated with or without TPEN (5 μM) for 3 h, and then MG-132 (25 μM) was added and incubated for 5 h. Total cell lysates were prepared with 1/10 of the cell lysates saved as input control. The rest were subjected to Ni-NTA-agarose beads pull-down. Both input and eluted proteins were analyzed by immunoblotting with indicated antibodies. *B*, inhibition of 19S proteasome cap subunit Rpn11 does not prevent MDMX from degradation. MCF-7 cells were treated with 5 μM TPEN in the absence or presence of capzimin dimer (2.5, 5, 10, or 20 μM), or capzimin dimer only (5 μM) for 8 h. *C*, knockdown 19S proteasome cap subunit Rpn2 does not prevent MDMX from degradation. MCF-7 cells were transfected with siRNAs targeting Rpn2 (siRpn2_1 and siRpn2_2). Forty-eight hours after transfection, the cells were treated with 5 μM TPEN for 5 h. Then ZnSO_4_ (5 μM) was added as indicated for 5 h. *D*, knockdown of the 19S proteasome cap subunit Rpn1 does not prevent MDMX from degradation. MCF-7 cells were transfected with an siRNA pool targeting Rpn1 (siRpn1). Forty-eight hours after transfection, the cells were treated with TPEN (5 μM) for 5 h. Then ZnSO_4_ (5 μM) was added as indicated for 5 h. All the immunoblotting experiments were repeated at least twice to ensure the reproducibility of the results.

MDMX degradation is controlled in part by MDM2-mediated ubiquitination and proteasome degradation (9). To examine whether TPEN induces MDMX ubiquitination, we found that while MG-132 treatment led to the accumulation of ubiquitinated MDMX species, however, no such accumulation was observed in the presence of TPEN, indicating a ubiquitin-independent mechanism (Fig. 3*A*). Similarly, ubiquitination of p53 reduced upon TPEN treatment. It was previously reported that zinc chelation by TPEN abolishes the E3 ligase function of MDM2 (12). As MDMX is a well-known MDM2 E3 ligase substrate, we pretreated MCF-7 cells with 5 μM TPEN and then cotreated cells with two E3 ligase inhibitors of MDM2 [HLI373 (34) and compound 1 (35)]. We found that both inhibitors cannot rescue the effect of TPEN on cellular levels of MDMX (Fig. S2*A*), confirming that MDM2-mediated MDMX ubiquitination does not account for TPEN-induced MDMX degradation.

As the 20S proteasome has been shown to degrade proteins that contain unstructured regions by a ubiquitin-independent mechanism (36), we speculated that zinc depletion could lead to MDMX misfolding, resulting in its 20S but not 26S proteasome-mediated degradation. To test this possibility, we cotreated MCF-7 cells with 5 μM TPEN along with an increased dosage of capzimin dimer (CZM), an inhibitor of the 19S proteasome cap subunit Rpn11. As CZM did not prevent MDMX from degradation, this suggested that 26S proteasome function is not required for MDMX degradation (Fig. 3*B*). Intriguingly, at higher concentrations, CZM treatment induced a similar effect as TPEN and both drugs displayed a synergistic effect on MDMX and p53 expression. Note that CZM is a quinoline-8-thiol derivative and its inhibition of Rpn11 activity could be through binding to the catalytic Zn^2+^ ion in the active site of Rpn11 (37). Therefore, it is not surprising that CZM may also function as a zinc chelator and facilitate 20S proteasome-mediated MDMX degradation. Furthermore, we transfected MCF-7 cells with siRNAs targeting the 19S proteasome cap subunits PSMD1 (non-ATPase regulatory subunit; a.k.a. Rpn2) or PSMD2 (non-ATPase regulatory subunit; a.k.a. Rpn1) and examined the effect of TPEN on cellular levels of MDMX in those cells. We found that TPEN retained the ability to induce MDMX degradation even though the basal levels of MDMX increased in siRpn1 and siRpn2-2 transfected cells (Fig. 3, *C* and *D*). Note that siRpn2-2-treated cells had higher basal levels of MDMX than siRpn2-1 or control siRNA-treated cells. The inconsistent effect might due to the off-target effect of one of the siRNAs. Alternatively, the knockdown of Rpn2 might need to reach a certain threshold level to block the 26S proteasome function toward MDMX as we noticed that siRpn2-2 had a higher efficiency to knockdown Rpn2. We would like to point out that cellular levels of p53 were elevated upon inhibition of 26S proteasome function as previously reported (38). Together, our results demonstrate that in the presence of TPEN, MDMX is targeted for 20S proteasome-mediated degradation in a ubiquitination-independent manner.

**Figure 4:**
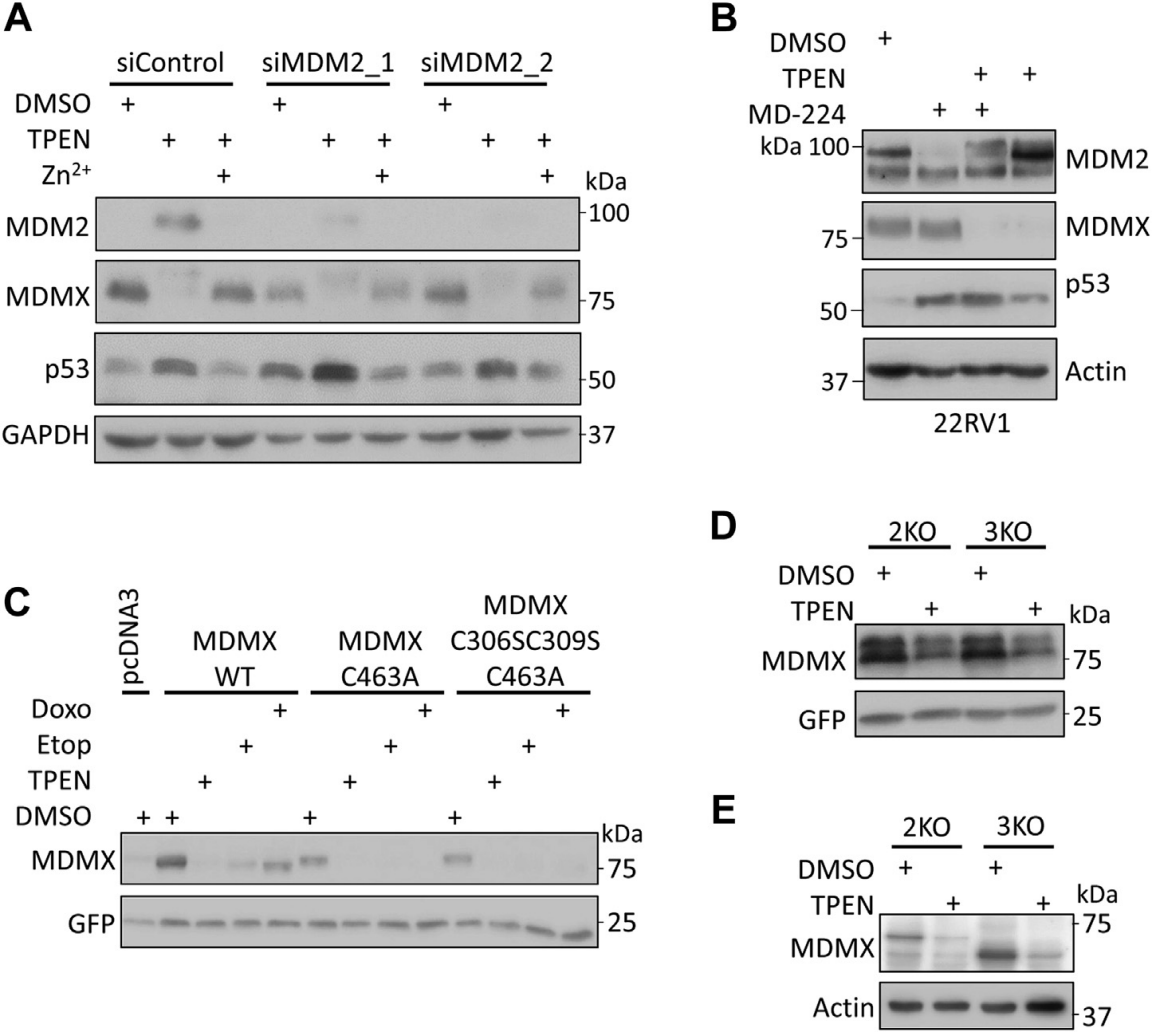
**TPEN-induced MDMX degradation is independent of MDM2.***A*, Depletion of MDM2 by siRNAs does not prevent TPEN-mediated MDMX degradation. MCF-7 cells were transfected with two different siRNAs targeting MDM2 (siMDM2_1 and siMDM2_2). Forty-eight hours later, the cells were incubated with 5 μM TPEN for 5 h, and then treated with or without 5 μM ZnSO_4_ for another 5 h (*B*) MDM2 PROTAC degrader MD-224 does not protect MDMX from TPEN-induced degradation. 22RV1 cells were pretreated with DMSO (control) and 30 nM MD-224 for 2 h, and then treated with DMSO (control) or 5 μM TPEN for 5 h. The cells were harvested and total cell lysates were analyzed by immunoblotting with indicated antibodies. *C*, MDMX mutants that cannot interact with MDM2 are sensitive to TPEN treatment. MCF-7 cells in 60-mm dishes were transfected with 0.75 μg of wild-type Myc-MDMX or Myc-MDMX mutants (C463A or C306SC309SC463A). Six hours after transfection, the cells were trypsinized and plated into four 35-mm dishes. After 18 h, the cells were treated with DMSO, TPEN (5 μM), etoposide (20 μM), or doxorubicin (10 μM) for 8 h (*D*) TPEN reduces levels of ectopically expressed MDMX in *mdm2*-null MEF cells. MEF 2KO (*p53*−/−, *mdm2*−/−) or 3KO (*p53*−/−, *mdm2*−/−, *mdmx*−/−) cells were transfected with Myc-MDMX plasmids (0.5 μg). GFP plasmids (0.05 μg) were also included as transfection control. Twenty-four hours after transfection, the transfected cells were treated with 5 μM TPEN for 8 h. MDMX proteins were analyzed by immunoblotting with anti-MDMX 8C6 antibody. *E*, TPEN reduces levels of endogenous MDMX in *mdm2*-null MEF cells. 2KO (*p53*−/−, *mdm2*−/−) or 3KO (*p53*−/−, *mdm2*−/−, *mdmx*−/−) MEF cells were treated with 5 μM TPEN for 8 h. Endogenous MDMX proteins were analyzed by immunoblotting with anti-MDMX 7A8 antibody. All immunoblotting experiments were repeated at least twice to ensure the reproducibility of the results.

As MDM2 has been found to associate with several subunits of the 19S proteasome regulatory particle and target p53, p21, and Rb for proteasome-dependent but ubiquitination-independent degradation, we assumed that the accumulated MDM2 upon TPEN treatment would still somehow facilitate the degradation of MDMX. Accordingly, we transfected MCF-7 cells with control siRNA or two different siRNAs, which target MDM2. Remarkably, TPEN induced MDMX degradation in cells treated with the MDM2 siRNAs to a similar extent as in cells treated with control siRNA (Fig. 4*A*). Similarly, TPEN induced MDMX degradation to a similar extent in MCF-7 cells with or without pretreatment of MD-224, an MDM2 PROTAC degrader (39) (Fig. S2*B*). Note that MD-224 failed to degrade MDM2 but induced cellular levels of p53 in MCF-7 cells. In 22RV1 cells, a prostate cancer cell line that MD-224 did degrade MDM2 under our experimental condition, and TPEN induced MDMX degradation with or without MD-224 treatment (Fig. 4*B*). In addition, we constructed two mutant forms of MDMX containing a mutation (C463A) that is known to be defective in MDM2 interaction (40, 41): (i) MDMX C463A that changes a key zinc coordinating Cys463 residue within the MDMX RING domain and (ii) a triply mutated MDMX (C306S/C309S/C463A) that is defective in zinc coordination both in the MDMX central zinc finger as well as in the C-terminal RING domain. We found that both MDMX mutants are sensitive to TPEN treatment (Fig. 4*C*), further supporting our conclusion that MDM2 may not be required for TPEN-mediated MDMX degradation. Moreover, we transfected a construct expressing human MDMX into mouse embryo fibroblasts (MEFs) that lack MDM2 and p53 expression, 2KO (*p53*−/−, *mdm2*−/−) (42) or fibroblasts that also lack MDMX (a.k.a. Mdm4), 3KO (*p53*−/−, *mdm2*−/−, *mdmx*−/−) (43). In both MEF strains we found that the levels of ectopically expressed human MDMX protein levels decreased upon TPEN treatment (Fig. 4*D*). Importantly, when we examined endogenously expressed MDMX (Mdm4) protein in 2KO (*p53*−/−, *mdm2*−/−) MEF cells, we found that it too is decreased to TPEN treatment (Fig. 4*E*). Note that TPEN treatment also reduced the cellular levels of a previously reported (44) truncated version of MDMX (Mdm4) in 3KO (*p53*−/−, *mdm2*−/−, *mdmx*−/−) MEF cells (Fig. 4*E*). Based on several lines of evidence, we conclude that MDM2 is not likely to be required for TPEN-mediated MDMX degradation.

### TPEN-induced MDMX reduction does not require MDMX phosphorylation at key serine residues

**Figure 5:**
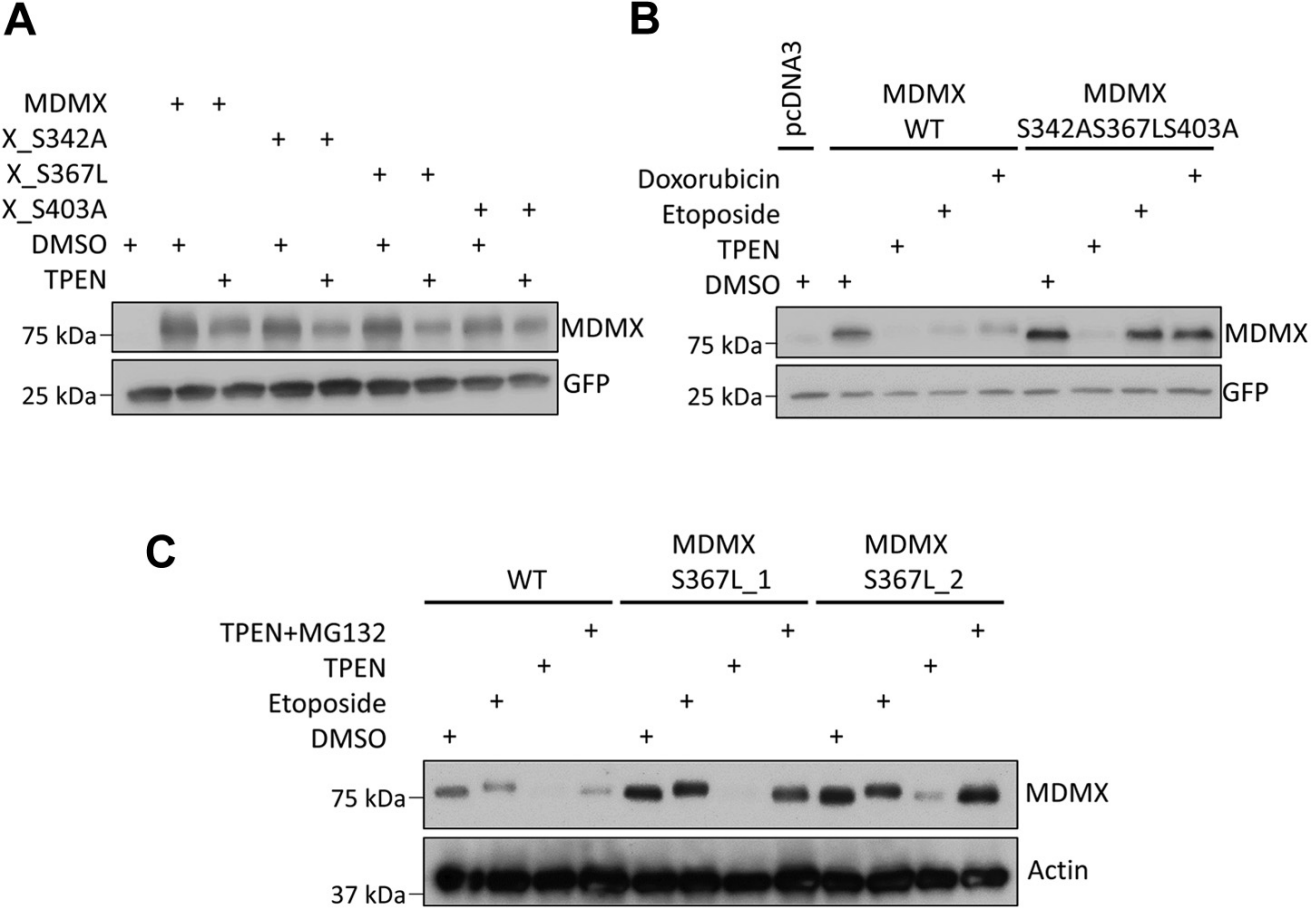
**TPEN-induced MDMX reduction does not require MDMX phosphorylation at Ser342, Ser367, or Ser 403.***A*, TPEN induces degradation of ectopically expressed MDMX variants S342A, S367L, or S403A. HEK293T cells were transfected with 0.15 μg of wild-type Myc-MDMX or Myc-MDMX variants S342A, S367L, or S403A. Twenty-four hours after transfection, the cells were treated with 5 μM TPEN for 8 h (*B*) MDMX mutant (S342AS367LS403A) is more resistant to etoposide or doxorubicin than TPEN treatment. MCF-7 cells in 60-mm dishes were transfected with 0.75 μg of wild-type Myc-MDMX or Myc-MDMX mutant (S342AS367LS403A). Six hours after transfection, the cells were trypsinized and plated into four 35-mm dishes. After 18 h, the cells were treated with DMSO, TPEN (5 μM), etoposide (20 μM), or doxorubicin (10 μM) for 8 h (*C*) TPEN induces endogenous MDMX S367L degradation. U2OS cells with endogenous MDMX S367L mutation were treated with TPEN (5 μM), TPEN (5 μM) plus MG132 (20 μM), or etoposide (15 μM) for 8 h. All the immunoblotting experiments were repeated at least twice to ensure the reproducibility of the results.

Stress-induced MDMX phosphorylation at several residues in its central domain has been shown to mediate MDMX nuclear translocation and subsequent degradation (32). We considered that phosphorylation events are needed for TPEN-mediated MDMX degradation and transfected HEK293T cells with different MDMX variants harboring mutation at Ser347, Ser367, or Ser403. Phosphorylation on these serine residues has been shown to be critical for DNA damage-induced MDMX degradation (32). As shown in Figure 5*A*, the mutant MDMX proteins were as sensitive to TPEN treatment as wild-type MDMX. Moreover, the MDMX protein carrying mutation at all these three residues was more resistant to etoposide or doxorubicin than TPEN treatment (Fig. 5*B*). Furthermore, using U2OS cell clones that express mutant MDMX (S367L) from the endogenous MDMX locus, we found that endogenous MDMX S367L is more resistant to etoposide than TPEN treatment (Fig. 5*C*). In line with these results, TPEN treatment induced cellular levels of phosphorylated H2AX, a marker for DNA double-strand breaks, although to a much lesser extent in comparison to etoposide, a topoisomerase II inhibitor, and chemotherapeutic agent (Fig. S3). Therefore, DNA damage-induced MDMX phosphorylation may not be involved in TPEN-mediated MDMX degradation. Nevertheless, we cannot rule out that phosphorylation at other residues contributes to MDMX degradation upon zinc depletion.

### The ion channel protein TRPM7 interacts with MDMX and regulates zinc depletion-induced MDMX degradation

**Figure 6:**
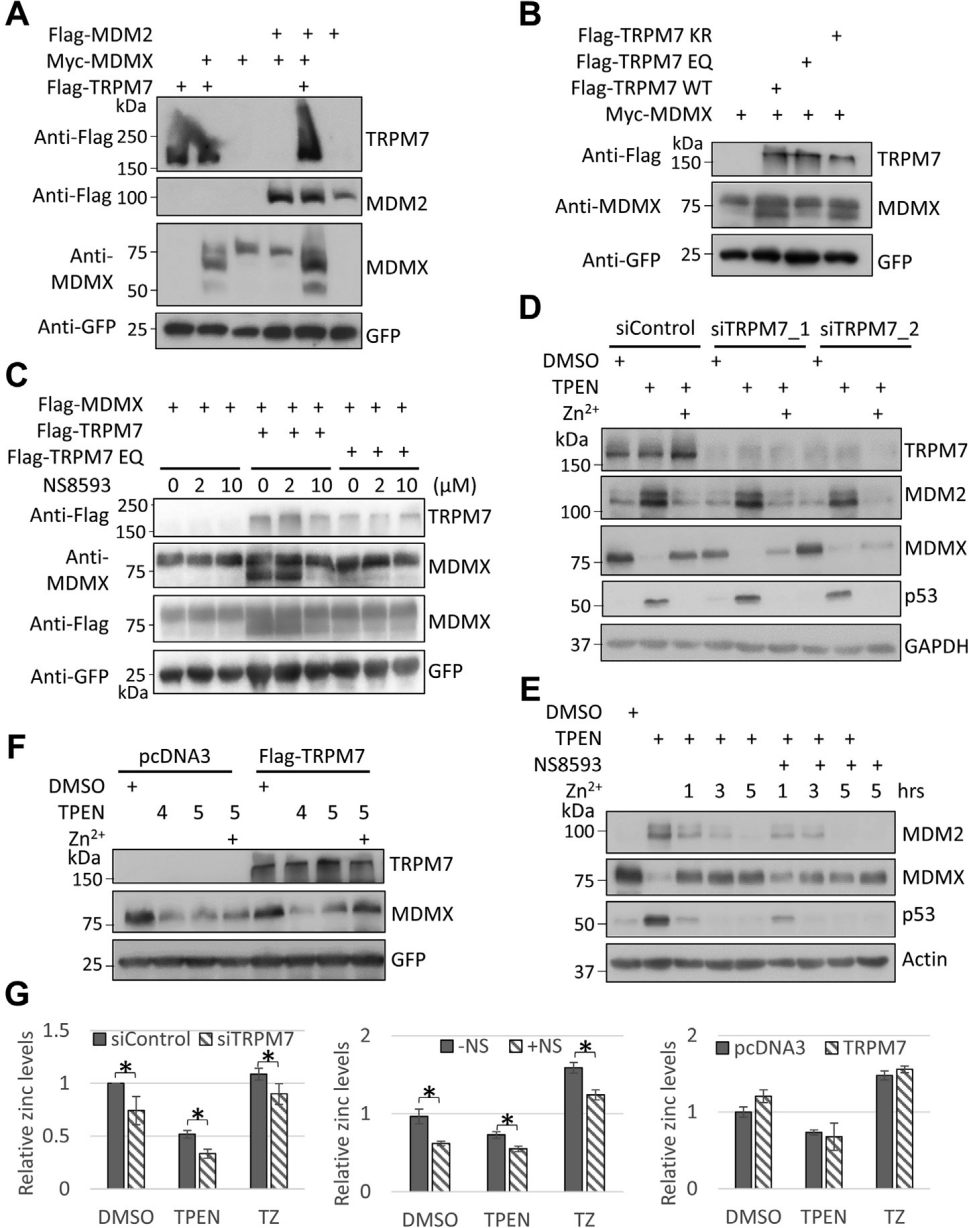
**The ion channel protein TRPM7 stabilizes ectopically expressed MDMX, induces the appearance of faster moving species of MDMX on SDS-PAGE, and regulates TPEN-induced MDMX degradation through its channel function.***A*, TRPM7 stabilizes ectopically expressed MDMX and induces the appearance of faster-moving species of MDMX on SDS-PAGE. HEK293T cells were transfected with combination of Myc-MDMX (0.15 μg), Flag-MDM2 (1 μg), and Flag-TRPM7 (1 μg) as indicated. Total cell lysates were analyzed 24 h after transfection. *B*, channel but not kinase function of TRPM7 is needed for TRPM7-induced appearance of faster-moving species of MDMX. HEK293T cells were transfected with Myc-MDMX (0.2 μg) and different Flag-TRPM7 variants (1 μg; wild-type, E1027Q, or K1646R) as indicated for 24 h (*C*) TRPM7 channel inhibitor NS8593 counteracts the effect of wild-type TRPM7 on MDMX stability and appearance of faster-moving species. HEK293T cells were transfected with Myc-MDMX (0.2 μg) and different Flag-TRPM7 variants (1 μg; wild-type, E1027Q, or K1646R) as indicated. Twenty-four hours after transfection, DMSO or NS8593 (2 or 10 μM) was added, and cells were incubated for another 12 h before harvesting. *D*, knockdown TRPM7 by siRNAs attenuates Zn^2+^-mediated recovery of MDMX in the presence of TPEN. MCF-7 cells were treated with control siRNA or two different siRNAs targeting TRPM7 (siTRPM7-1 and siTRPM7-2). Forty-eight hours later, the cells were treated with 5 μM TPEN for 5 h and then incubated with or without 5 μM ZnSO_4_ for another 5 h (*E*) TRPM7 channel inhibitor NS8593 attenuates Zn^2+^-mediated recovery of MDMX in TPEN-treated MCF-7 cells. MCF-7 cells were treated with 5 μM TPEN for 5 h and then 5 μM ZnSO_4_ was added in the absence of presence of 50 μM NS8593. The cells were harvested at 1, 3, or 5 h after ZnSO_4_ and NS8593 treatment. *F*, overexpression of TRPM7 facilitates Zn^2+^-mediated recovery of MDMX in TPEN-treated HEK293T cells. HEK293T cells were plated in 60 mm dishes and transfected with Flag-TRPM7 variants (2 μg). Twenty-four hours after transfection, cells were trypsinized and replated into 35 mm dishes. Twenty-four hours after replating, the cells were treated with DMSO, 4 μM, or 5 μM TPEN for 5 h. Then 5 μM ZnSO_4_ was added into one of TPEN-treated dish. One hour later, the cells were harvested and total cell lysates were prepared and subjected to immunoblotting with anti-Flag, anti-MDMX, and anti-GFP antibodies. *G*, TRPM7 modulates intracellular levels of Zn^2+^. HEK293T cells transfected with control siRNA or siRNA targeting TRPM7 (siTRPM7_2) (*left panel*; 50 nM each; 48 h after transfection) or HEK293T cells transfected with pcDNA3, Flag-TRPM7 (*right panel*; 5 μg each; 24 h after transfection), or MCF-7 cells (*middle panel*) were loaded with Zinpyr-1 (5 μM) for 30 min at 37 °C. Then the cells (for HEK293T, 4 × 10^4^ cells; for MCF-7, 3 × 10^4^ cells) were plated, treated as indicated, and the intracellular zinc levels were measured and calculated as described in Experimental procedures. The relative zinc levels were calculated by normalizing to the measurements of DMSO-treated samples. The results were expressed as means ± sd of three independent experiments. The *asterisk* indicates statistical significance (*p* value <0.05). All the immunoblotting experiments had been repeated at least twice to ensure the reproducibility of the results.

In a mass-spectrometry-based proteomics analysis, we identified TRPM7, an ion channel protein, as a potential MDM2/MDMX heterodimer interacting protein (Fig. S4, *A*–*C*). Using coimmunoprecipitation assay with ectopically expressed proteins, we confirmed its interaction with MDMX (Fig. S4*D*). Intriguingly, when we ectopically expressed TRPM7 with MDMX in the presence or the absence of MDM2, we found that TRPM7 coexpression stabilized MDMX and induced the appearance of a more rapidly migrating MDMX polypeptide on SDS-PAGE (Fig. 6*A*). Since TRPM7 is a bifunctional protein with both ion channel and α-type kinase function (20), we introduced either a channel-defective mutation E1047Q (45) or a kinase-dead mutation K1646R (46) into TRPM7 and examined its effect on cotransfected MDMX. As shown in Figure 6*B*, the TRPM7 E1047Q but not the TRPM7 K1646R was unable to stabilize MDMX or induce the appearance of the faster moving species of MDMX on SDS-PAGE. Moreover, NS8593, a specific TRPM7 channel inhibitor (47), counteracted the effects of wild-type TRPM7 on MDMX (Fig. 6*C*). Together, our results reveal that the channel function of TRPM7 is critical for TRPM7-induced MDMX stabilization and the appearance of faster moving species of MDMX on SDS-PAGE.

To further test if TRPM7 is involved in TPEN-mediated MDMX degradation, MCF-7 cells were transfected with control siRNA or two different siRNAs that target TRPM7. Forty-eight hours later, cells were pretreated with 5 μM TPEN and then 5 μM ZnSO_4_ was added. As shown in Figure 6*D*, Zn^2+^-mediated recovery of MDMX in the presence of TPEN is attenuated upon the depletion of TRPM7.

In line with the data obtained with TRPM7 knockdown, NS8593 attenuated the effect of Zn^2+^ on TPEN-induced MDMX degradation (Fig. 6*E*). On the other hand, we found that in TPEN-treated HEK293T cells, overexpression of TRPM7 facilitates the Zn^2+^-mediated recovery of MDMX (Fig. 6*F*). Note that the observed effects of TRPM7 knockdown, inhibitor treatment, or overexpression on MDMX most likely happened at a posttranscriptional level as the mRNA levels of MDMX remained fairly constant under the experimental condition (Fig. S5). In addition, we monitored the intracellular zinc levels with a zinc sensor Zinpyr-1 (48). As shown in Figure 6*G* (cell-based microplate assays) and Figure S6 (flow cytometer measurements), TRPM7 knockdown or inhibition by NS8593 significantly decreased intracellular levels of zinc. We also observed that TRPM7 overexpression increased intracellular levels of zinc, although the result was not statistically significant under our experimental condition (Fig. 6*G*). Together, our data indicated that TRPM7 is involved in Zn^2+^-mediated recovery of MDMX in TPEN-treated cells, which is most likely through its channel function.

### MDMX overexpression partially rescues the inhibitory effect of TPEN but not TRPM7 inhibition on cancer cell growth

**Figure 7:**
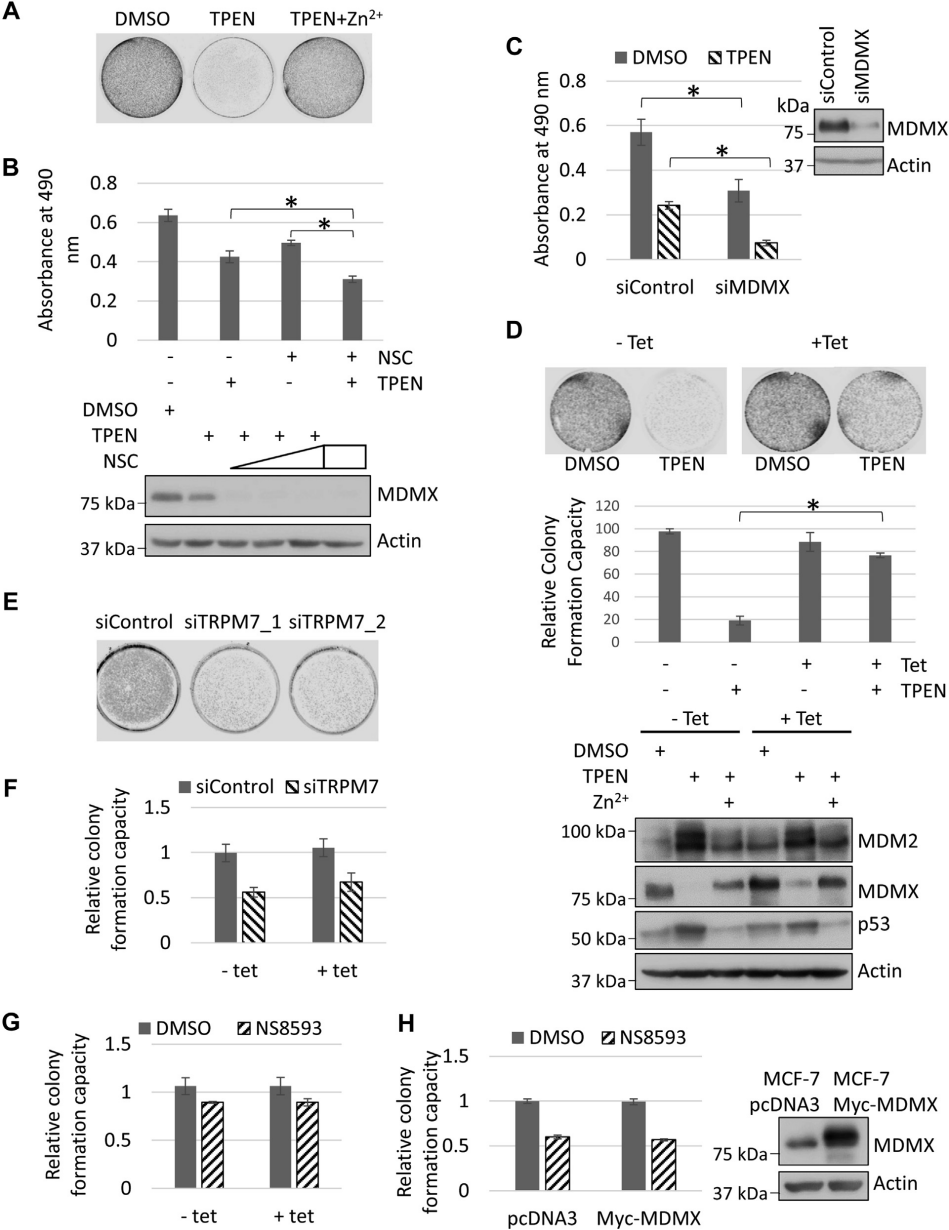
**MDMX overexpression partially reverses the inhibitory effect of TPEN but not TRPM7 inhibition on MCF-7 cell growth.***A*, TPEN treatment inhibits MCF-7 cell growth in a zinc-dependent manner. 5 × 10^4^ MCF-7 cells were plated on 35 mm dishes. Twenty-four hours later, the cells were treated with DMSO, TPEN (5 μM), or TPEN plus ZnSO_4_ (5 μM each) for 24 h. Fresh DMEM medium was then added into each dish and replaced every other day. The cells were fixed and stained 5 days after treatment. *B*, NSC207895 further inhibits TPEN-repressed MCF-7 cell proliferation. *Top panel*: 1 × 10^4^ MCF-7 cells were plated on 96-well plates and treated in triplicates with DMSO, TPEN (5 μM), NSC207895 (2.5 μM), or TPEN (5 μM) plus NSC207895 (2.5 μM) for 8 h. Cell proliferation was measured as described in Experimental procedures. The results were expressed as means ± sd of three independent experiments. The *asterisk* indicates statistical significance (*p* value <0.05). Bottom panel: MCF7 cells were treated with DMSO, TPEN (5 μM), NSC207895 (NSC; 5 μM), or TPEN (5 μM) plus increasing dosages of NSC207895 (NSC; 1, 2.5, or 5 μM) for 8 h. The cells were harvested and total cell lysates were prepared and subjected to immunoblotting with anti-MDMX and anti-actin antibodies. *C*, depletion of MDMX by siRNA enhances the inhibitory effect of TPEN on MCF-7 cell proliferation. MCF-7 cells were transfected with control siRNA or siRNA targeting MDMX (siMDMX). Eighteen hours later, the cells were trypsinized, counted, and plated (6 × 10^3^ cells for each well) on 96-well plates in triplicates. After 24 h, cells were treated with DMSO or TPEN (5 μM) for 8 h. Cell proliferation was measured as described in Experimental procedures. The results were expressed as means ± sd of three independent experiments. The *asterisk* indicates statistical significance (*p* value <0.05). Knockdown of MDMX by siRNA in MCF-7 cells was confirmed by western blot analysis. *D*, MDMX overexpression partially rescues the inhibitory effect of TPEN on MCF-7 cells. *Top* and *middle panel*: 5 × 10^4^ MCF-7 cells with tetracycline-inducible MDMX overexpression were plated on 6-well plate with or without 2.25 μg/ml of tetracycline and treated with DMSO or 5 μM TPEN for 24 h. Fresh medium was then added and replaced every other day. Five days later, the cells were fixed and stained. Relative colony formation capacity was quantified as described in Experimental procedures based on three independent experiments. The *asterisk* indicates statistical significance (*p* value <0.05). *Bottom panel*: MCF7 cells with tetracycline inducible MDMX overexpression were plated on 6-well plate with or without 2.25 μg/ml of tetracycline. Twenty-four hours later, the cells were treated with DMSO or 5 μM TPEN. 5 μM ZnSO4 was added in one of the TPEN-treated cells 5 h later. After another 5 h incubation, the cells were harvested and total cell lysates were analyzed by immunoblotting with indicated antibodies. *E*, knockdown of TRPM7 inhibits MCF-7 cell growth. MCF-7 cells were treated with control siRNA or siRNAs targeting TRPM7 (siTRPM7_1 and siTRPM7_2). Twenty-four hour after transfection, 2 × 10^4^ cells were plated in 6-well plate and then fixed and stained 1 week later. *F*, MDMX overexpression does not rescue TRPM7 ablation-mediated cell growth inhibition in a tetracycline-inducible MDMX overexpression MCF-7 system. 5 × 10^4^ MCF7 cells with tetracycline-inducible MDMX overexpression were plated on 6-well plate in triplicates with or without 2.25 μg/ml of tetracycline. Twenty-four hours later, the cells were transfected with control siRNA or siRNA targeting TRPM7 (siTRPM7_2). Fresh medium was added 6 h after treatment and replaced every other day. Five days later, the cells were fixed and stained with crystal violet (0.05% in 20% ethanol). Relative colony formation capacity was quantified as described in Experimental procedures based on three independent experiments. No statistical significance was observed. *G*, TRPM7 inhibitor NS8593 does not affect cell growth for tetracycline-inducible MDMX overexpression MCF7 cell system. 5 × 10^4^ MCF7 cells with tetracycline-inducible MDMX overexpression were plated on 6-well plate per well in triplicates with or without 2.25 μg/ml of tetracycline in the absence or presence of NS8593 (30 μM). Medium was replaced every other day. Five days later, the cells were fixed and stained with crystal violet (0.05% in 20% ethanol). Relative colony formation capacity was quantified as described in Experimental procedures based on three independent experiments. No statistical significance was observed. *H*, TRPM7 inhibitor NS8593 does not affect cell growth for MDMX overexpression stable MCF7 cell system. 5 × 10^4^ MCF7-pcDNA3 and MCF7-Myc-MDMX cells were plated on 6-well plate in triplicates in the absence or presence of NS8593 (30 μM). Medium was replaced every other day. Five days later, the cells were fixed and stained with crystal violet (0.05% in 20% ethanol). Relative colony formation capacity was quantified as described in Experimental procedures based on three independent experiments. No statistical significance was observed. MCF7-Myc-MDMX cells expressing higher levels of MDMX in comparison to MCF-pcDNA3 cells were confirmed by western blot analysis. All the immunoblotting experiments were repeated at least twice to ensure the reproducibility of the results.

TPEN treatment inhibited MCF-7 cell growth on a colony formation assay, whereas the addition of zinc completely abolished the inhibitory effect of TPEN (Fig. 7*A*). TPEN treatment also repressed MCF-7 cell growth in a cell proliferation assay (Fig. 7*B*). NSC207895, an MDMX inhibitor that blocks the MDMX promoter and inhibits the MDMX expression (49), enhanced the inhibitory effect of TPEN on MCF-7 cell proliferation (Fig. 7*B*). Similar results were obtained using MCF-7 cells treated with control siRNA or siRNA targeting MDMX in the absence or presence of TPEN (Fig. 7*C*). On the other hand, using MCF-7 cells with tetracycline-inducible MDMX overexpression, we observed that the cells growing in the presence of tetracycline (a.k.a. with MDMX overexpression) were more resistant to the TPEN treatment in the colony formation assay (Fig. 7*D*). Together, these results indicated that MDMX protects MCF-7 cells from zinc depletion-mediated cell growth inhibition.

Previously it was reported that downregulation of TRPM7 in human cancer cells impairs cell proliferation, migration, and invasion (23). As our data suggested that TRPM7 actively regulates cellular levels of MDMX, presumably through its channel function on intracellular zinc concentration modulation, we reasoned that MDMX overexpression might counteract the effect of TRPM7 inhibition on MCF-7 cells. Consistent with previous reports, depletion of TRPM7 using two different siRNAs led to inhibition of MCF-7 cell growth in a colony formation assay (Fig. 7*E*). However, using MCF-7 cells with tetracycline-inducible MDMX overexpression inhibition of TRPM7 with either siRNAs (Fig. 7*F*) or NS8593 (Fig. 7*G*), we did not observe a cell growth difference for the cells with or without MDMX overexpression. Similarly, no cell growth difference was observed between MCF-7-Myc-MDMX cells with stably expressed Myc-MDMX and MCF-7-pcDNA3 control cells in response to NS8593 treatment (Fig. 7*H*). Therefore, under our experimental conditions, MDMX overexpression does not rescue TRPM7 inhibition-mediated growth of MCF-7 cells.

### MDMX overexpression partially rescues the inhibitory effect of TRPM7 inhibition on cancer cell migration

**Figure 8:**
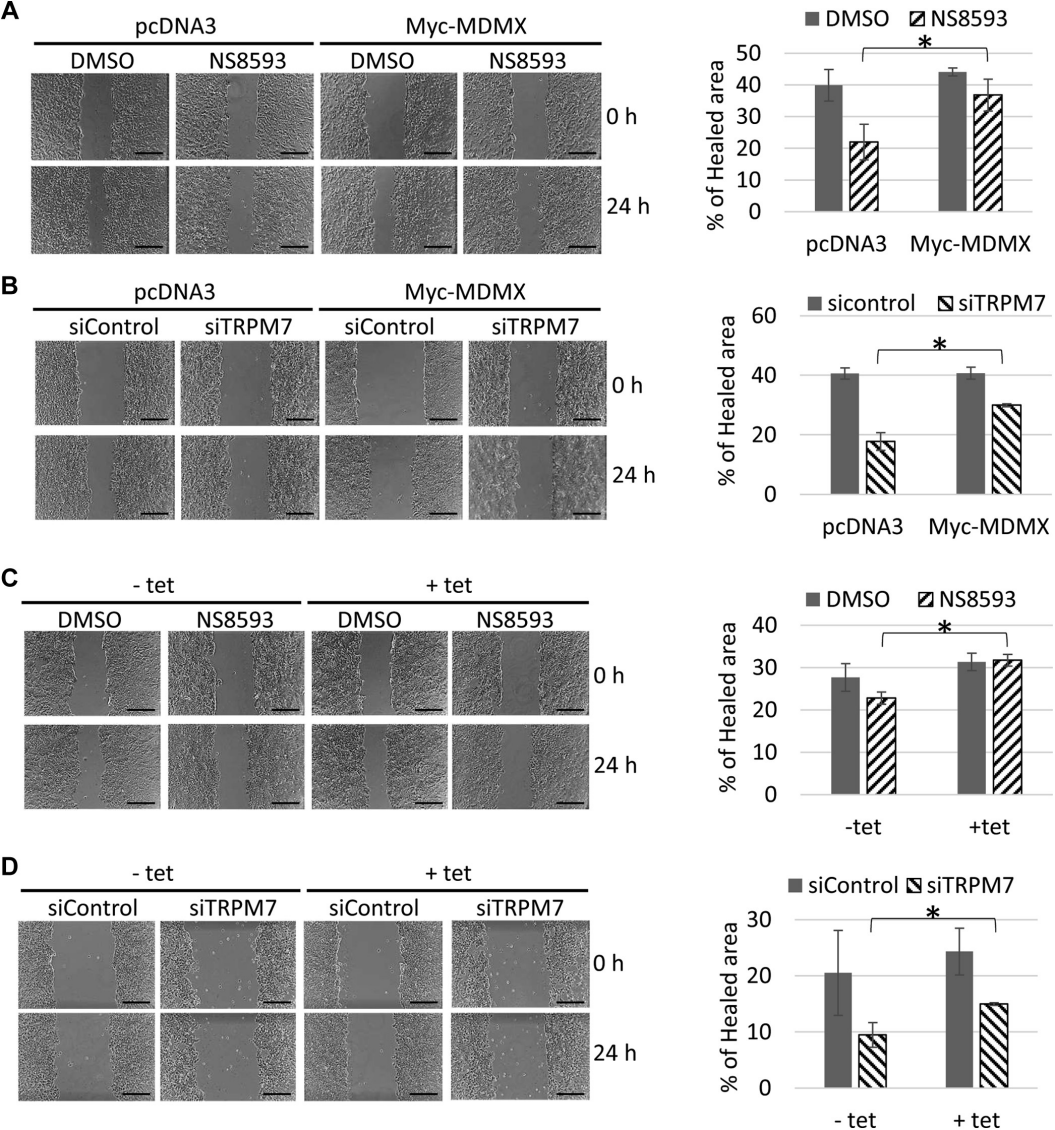
**MDMX overexpression partially reverses the inhibitory effect of TRPM7 inhibition on MCF7 cell migration.***A*, MDMX overexpression partially rescues cell migration repression mediated by NS8593 in MCF-7 cells with stably expressed MDMX. MCF-7-pcDNA3 and MCF-7-Myc-MDMX cells were plated on 6-well plate to reach ∼90% confluence. The cells were starved with DMEM plus 0.5% FBS for 24 h. After that, the cells were scratched and incubated in DMEM plus 0.5% FBS with DMSO or NS8593 (30 μM). Images were taken at 0 or 24 h after scratch (Scale bar = 500 μm). Percentage of healed wound area at 24 h related to 0 h time point was graphed as described in Experimental procedures. The *asterisk* indicates statistical significance (*p* value <0.05). *B*, MDMX overexpression partially rescues cell migration repression mediated by TRPM7 ablation in MCF-7 cells with stably expressed MDMX. MCF-7-pcDNA3 and MCF-7-Myc-MDMX cells were plated on 60 mm dishes for 24 h and then transfected with control siRNA or siRNA targeting TRPM7 (siTRPM7_2). Six hours after transfection, 2 × 10^6^ cells were plated in each well of a 6-well plate to reach ∼90% confluence. The cells were starved with DMEM plus 0.5% FBS for 24 h and then scratched. Images were taken at 0 or 24 h after scratch (Scale bar = 500 μm) and analyzed as described in Experimental procedures. The *asterisk* indicates statistical significance (*p* value <0.05). *C*, MDMX overexpression partially reverses cell migration repression mediated by NS8593 in MCF7 cells with tetracycline-inducible MDMX overexpression. MCF7 cells with tetracycline-inducible MDMX overexpression were plated on 6-well plate with or without 2.25 μg/ml of tetracycline to reach ∼90% confluence. The cells were starved with DMEM plus 0.5% FBS for 24 h. After that, they were scratched and incubated in DMEM plus 0.5% FBS with DMSO or NS8593 (30 μM). Images were taken at 0 or 24 h after scratch (Scale bar = 500 μm) and analyzed as described in Experimental procedures. The *asterisk* indicates statistical significance (*p* value <0.05). *D*, MDMX overexpression partially reverses cell migration repression mediated by TRPM7 knockdown in MCF7 cells with tetracycline-inducible MDMX overexpression. MCF7 cells with tetracycline-inducible MDMX overexpression were plated on 60 mm dishes for 24 h and then transfected with control siRNA or siRNA targeting TRPM7 (siTRPM7_2). Six hours after transfection, both cells were trypsinized and counted. 2 × 10^6^ cells were plated in each well of a 6-well plate to reach ∼90% confluence. The cells were starved with DMEM plus 0.5% FBS for 24 h. After that, they were scratched and images were taken at 0 or 24 h after scratch (Scale bar = 500 μm) and analyzed as described in Experimental procedures. The *asterisk* indicates statistical significance (*p* value <0.05).

Since MCF-7 is a noninvasive line naturally, it is not suitable for a transwell migration assay. We then assessed MCF-7 cell migration with a wound-scratch assay. As shown in Figure 8, we found that NS8593 treatment inhibited MCF-7 cell migration. Interestingly, MCF-7 cells with stably expressed MDMX (Fig. 8*A*) or MCF-7 cells with tetracycline-induced MDMX overexpression (Fig. 8*C*) were partially resistant to this inhibitory effect in comparison to control cells. Similarly, depletion of TRPM7 using siRNA inhibited MCF-7 cell migration while MCF-7 cells with stably expressed MDMX (Fig. 8*B*) or MCF-7 cells with tetracycline-induced MDMX overexpression (Fig. 8*D*) were partially resistant to this inhibitory effect in comparison to control cells. Together, our data indicated that TRPM7 may function through its regulation of MDMX to affect cell migration during tumorigenesis.

## Discussion

Dysregulation of metal homeostasis contributes to cancer development and several chelators targeting metal ions such as iron and copper are currently in clinical trials for cancer treatment (50, 51). As metal ions are vital for cellular functions at chemical, molecular, and biological levels, different mechanisms may be employed by various chelators for their antitumor function. In particular, several iron chelators have been shown to stabilize p53, although the stabilized p53 may not be transcriptionally active. The antitumor effect of these iron chelators may be in part *via* p21 through a p53-independent pathway or may involve the p53 family member p73 (51, 52, 53, 54). Relevant to this study, zinc chelation has also been shown to stabilize p53, most likely through the inhibition of E3 ligase activity of MDM2. Similarly, such stabilized p53 is not transcriptionally active due to the disruption of the zinc-containing DNA-binding domain of p53 (55). Here, we report that zinc chelation by TPEN results in MDMX degradation in a ubiquitination-independent and 20S proteasome-dependent manner. Moreover, we identified TRPM7, a zinc-permeable ion channel, as a novel MDMX-interacting protein. TRPM7 inhibition by siRNAs or channel inhibitor NS8593 attenuates while TRPM7 overexpression facilitates the recovery of MDMX upon adding back of zinc to TPEN-treated cells. Most importantly, we found that TRPM7 inhibition suppresses breast cancer MCF-7 cell migration, which can be partially rescued by the overexpression of MDMX. In all, our data indicate that TRPM7 regulates cellular levels of MDMX in part by modulating intracellular zinc concentration. As such our findings with TRPM7 could suggest a therapeutic target for combinational cancer treatment.

In breast cancer patients, it has been reported that zinc levels are lower in serum but are higher in breast cancer tissue than in normal breast tissue (56). It is intriguing that breast cancer tissue has a significantly high uptake of zinc, and it is unclear if these changes in serum and tissue zinc concentrations contribute to the initiation, promotion, or progression of breast cancer, or whether if they are the effects of malignant transformation. We observed that cellular levels of MDMX correlate with the intracellular zinc concentration. MDMX overexpression partially rescues the inhibitory effect of zinc depletion on cell growth, indicating that zinc deficiency may impose selection pressure for cells with MDMX amplification or overexpression during breast cancer development.

Zinc affects the cellular levels of MDMX at the posttranscriptional level. Since MDMX is a zinc-containing protein with two zinc coordination motifs, we anticipated that zinc depletion leads to partially unstructured MDMX, which is subjected to 20S proteasome-mediated degradation. It is intriguing that MDM2, a well-known E3 ubiquitin ligase for both p53 and MDMX (9) that has also been shown to directly interact with the 20S proteasome and facilitate 20S proteasome-mediated p53 (57) or Rb (15) degradation, is not involved in zinc depletion-mediated MDMX degradation. Since MDM2 and MDMX bind to each other through their RING-finger domains, zinc depletion may impair their interaction by affecting the RING-finger structure. Further investigation will be needed to test if MDMX can bind to 20S proteasome by itself or if other unknown proteins are required for its 20S proteasome-mediated degradation.

Zinc transporters, zinc-permeable ion channels, and zinc-sequestering metallothioneins have been shown to be critical for intracellular zinc homeostasis. Deregulation of their function contributes to tumorigenesis (58, 59). Multiple zinc transporters (such as ZnT2, ZIP6/LIV1, ZIP7 and ZIP10), zinc-permeable ion channels (such as TRPC6, TRPM7, and TRPV6), as well as metallothionein were reported to be overexpressed in breast cancer, some of which are associated with breast cancer metastases and poor prognosis (60). Relatedly, TRPM7 is abnormally overexpressed in various cancer cells including breast cancer (23) and knockdown of TRPM7 suppresses breast cancer cell migration and invasion (61).

We had identified TRPM7 as a novel MDMX interacting protein and found that it both stabilizes MDMX and induces faster moving species of the MDMX on SDS-PAGE when the two proteins are ectopically coexpressed. Although the functional importance of these faster moving species of MDMX remains unknown, we found that the channel domain but not the kinase domain of TRPM7 is essential for the observation under the experimental condition. Importantly, we revealed that overexpression of TRPM7 facilitates but TRPM7 ablation or NS8593 treatment inhibits the recovery of cellular levels of MDMX after adding back zinc to TPEN-treated cells. Therefore, TRPM7 actively regulates cellular levels of MDMX, most likely through its zinc permeable channel function. Intriguingly, we observed a full reverse of MDM2 and p53 expression but an incomplete recovery of MDMX expression under the experimental condition. We hypothesize that the recovery of zinc-coordinating RING domain upon addition of the zinc supplement will facilitate the formation of MDM2 homo-oligomers or even MDM2-MDMX hetero-oligomers, enhancing the MDM2 E3 ligase activity and resulting in rapid degradation of p53 and MDM2. On the other hand, TRPM7 may affect the recovery of MDMX upon zinc supplement after TPEN treatment through two mechanisms: (i) mediating the zinc influx so that MDMX can coordinate zinc for a proper structure to avoid degradation; (ii) interacting with MDMX and protecting it from degradation. The two mechanisms may function in concert as TRPM7 may increase the local zinc concentration around MDMX through its interaction with MDMX, allowing a more efficient recovery of MDMX folding. The recovery of cellular levels of MDMX may have a slower kinetics due to TRPM7 knockdown. In addition, the recovered MDMX may be targeted for degradation by MDM2. Furthermore, we demonstrated that suppression of MCF-7 cell migration upon the inhibition of TRPM7 can be partially rescued by MDMX overexpression. Based on these findings, we speculate that overexpression of TRPM7 in breast cancer cells raises their intracellular zinc concentration, which, in turn, increases cellular levels of MDMX to promote cancer cell migration. As the cleaved C-terminal kinase domain of TRPM7 has other activities in the nucleus (26), it is very likely that other mechanisms are employed by TRPM7 to promote breast cancer progression. Future investigations will be needed to explore those mechanisms.

TRPM7 is not unique among proteins that are relevant to zinc biology in regard to breast cancer. Other zinc-permeable ion channels, zinc transporters, as well as metallothioneins may employ similar mechanisms to influence breast cancer development by modulating the cellular levels of zinc and MDMX levels. Indeed, when we knocked down ZIP7, a zinc transporter that is overexpressed in breast cancer, we observed similarly attenuated MDMX recovery in TPEN-treated MCF-7 cells upon adding back zinc (Fig. S7). Therefore, modulating the intracellular zinc concentration may be an effective therapeutic strategy for cancer treatment. Depending on cellular levels of MDMX and TRPM7 as well as other proteins involved in zinc homeostasis, therapeutic strategies and zinc supplementation guidelines have potential for development as cancer therapeutics, diagnosis, or prevention.

## Experimental procedures

### Cell culture and plasmids

Hek293T, U2OS, MCF-7, PC3 and 22RV1, MEF 2KO (*p53*−/−, *mdm2*−/−), and MEF 3KO (*p53*−/−, *mdm2*−/−, *mdmx*−/−) cells were cultured in DMEM medium with 10% FBS (Fetal Bovine serum, Gemini Bio-products) and 1% penicillin-streptomycin (Gibco) at 37 °C, 5% CO_2_. MEFs were provided by Dr Guillermina Lozano (MD Anderson Cancer Center). LNCaP cells were cultured in RPMI medium with 10% FBS (Fetal Bovine serum, Gemini Bio-products) and 1% penicillin streptomycin (Gibco) at 37 °C, 5% CO_2_. For zinc-deficient DMEM medium, FBS was incubated overnight with 5% Chelex 100 (Bio-Rad) before supplemented (10%) into DMEM medium. MCF-7-pcDNA3 and MCF-7-Myc-MDMX cell lines were constructed by transfecting pcDNA3 or Myc-MDMX plasmid into MCF-7 cells and then selecting G418 (1 mg/ml) resistant clones for 2 weeks. Pooled cell clones were maintained in DMEM with 250 μg/ml G418. MCF-7 with tetracycline-inducible MDMX cell line was obtained from Dr Xinbin Chen (University of California Irvine) and was maintained in DMEM with 4 μg/ml Blasticidin and 25 μg/ml Zeocin. U2OS MDMX S367L mutant cells were generated using CRISPR/Cas9 genome-editing technology (62) as described in supporting information.

HA-p53, Flag-MDM2, Myc-MDMX, and His-Ubiquitin were described previously (63). Flag-TRPM7 was a kind gift from Dr Clapham (Howard Hughes Medical Institute). Myc-MDMX variants (S342A, S367L, S403A, D361A, S342AS367LS403A, C463A, and C306SC309SC463A) and Flag-TRPM7 variants (E1047Q and K1646R) were constructed by site-directed mutagenesis. All the mutations were confirmed by Sanger sequencing.

### Antibodies and reagents

Commercially obtained antibodies used were as follows: MDMX (A300–287A, Bethyl; or 8C6, Millipore), actin (C-4, Santa Cruz Biotechnology), Flag (M2, Sigma), HA (HA.11, Covance), TRPM7 (A302-700A, Bethyl), Parp (9542; Cell Signaling Technology), LC3B (NB100-2220; Novus), Rpn1 (sc-271775, Santa Cruz Biotechnology), Rpn2 (A303–851A, Bethyl), and GFP (B-2, Santa Cruz Biotechnology). Mouse monoclonal antibodies against human p53 (DO-1) and MDM2 (3G5, 4B11, 5B10), MDMX (7A8, kindly provided by Dr Jiandong Chen, Moffitt Cancer Center) were used as supernatants from hybridoma cultures. Reagents used in this study were as follows: TPEN (N, N, N′, N′ -tetrakis (2-pyridinylmethyl) - 1,2-ethanediamine), MG132, CaCl_2_, MgCl_2_, and ZnSO_4_ from Sigma; NS8593, bispicen, chloroquine, bafilomycin A1, capzimin dimer, Z-VAD-FMK, and NSC207895 from Calbiochem; Zinpyr-1 from Santa Cruz Biotechnology.

### Transfections

Plasmid transfections were performed using PEI (Polysciences; cat# 23966-2) for HEK293T cells and Lipofectamine 2000 (Thermo Fisher Scientific) for MCF-7 cells. siRNA transfections in MCF-7 cells were performed using Dharmafect 1 (Dharmacon) using 50 nM of control siRNA (Allstar negative control siRNA; Qiagen), siMDMX (Hs_MDM4_4 FlexiTube siRNA; Qiagen), siRpn1 (sc-62898; Santa Cruz Biotechnology), or siRNA targeting TRPM7, MDM2 or Rpn2. The target sequences are: 5′-TTAATGTATCTACCGTCAGGG-3′ (siTRPM7_1), 5′-GAGTATTTCATGGCAAGAC-3′ (siTRMP7_2), 5′-AAGCCATTGCTTTTGAAGTTA-3′ (siMDM2_1), 5′- AAGGAATAAGCCCTGCCCA-3′ (siMDM2_2). 5′-GTCTAGATGATCACAAGTA-3′ (siRpn2_1) and 5′-GGGTGTAATTCATAAGGGT-3′ (siRpn2_2).

### RNA extraction and quantitative RT-PCR analysis

RNA was extracted using a Qiagen RNeasy mini-kit, and cDNA was synthesized with the QuantiTect reverse transcription kit (Qiagen). Samples were analyzed by quantitative real-time PCR on a Bio-Rad CFX 96 using PowerUp SYBR Green (Thermo Fisher Scientific). RNA expression was normalized to RPL32 mRNA expression. Relative levels were calculated by the comparative Ct method (ΔΔCT method). The results are expressed as means ± sd of four experiments. Primer sequences are: RPL32, 5′-TTCCTGGTCCACAACGTCAAG-3′(Forward) and 5′-TGTGAGCGATCTCGGCAC-3′ (Reverse); MDMX, 5′-GCAAGAAATTTAACTCTCCAAGCAA-3′ (Forward) and 5′-CTTTGAACAATCTGAATACCAATCCTT-3′ (Reverse).

### *In vivo* ubiquitination assay

*In vivo* ubiquitination assays were carried out using a His-Ub construct. After cells were harvested, a Ni-NTA pull-down assay was carried out as previously described (63). Eluted proteins were analyzed by Western blotting with anti-MDMX or anti-p53 antibody.

### Intracellular Zn^2+^ measurements

Zinpyr-1 (Santa Cruz Biotechnology) was used to detect free intracellular in live cells. Cells were incubated with 5 μM Zinpyr-1 in DMEM for 30 min at 37 °C. Then the cells were washed twice with 1× PBS, trypsinized, and resuspended. The cell numbers were counted, and the cell suspension was adjusted to a final concentration of 4 × 10^5^ (for HEK293T cells) or 3 × 10^5^ (for MCF-7 cells) cells/ml. In total, 100 μl of diluted cells was aliquoted into 96-well plates. For HEK293T cells, 100 μl of DMEM containing DMSO, TPEN (10 μM), or TPEN plus ZnSO_4_ (10 μM each) was added in triplicates into the cell-containing wells. For MCF-7 cells, 100 μl of DMEM containing DMSO, NS8593 (100 μM), TPEN (10 μM), or ZnSO_4_ (10 μM) was added in triplicates alone or in combination as indicated into the cell-containing wells. The fluorescence intensity was measured on a TECAN SPARK Microplate Reader (Tecan) after the plates were incubated for 30 min at 37 °C. The cells were excited at a wavelength of 488 nm (bandwidth of 20 nm), and the emission was measured at a wavelength of 535 nm (bandwidth of 20 nm). The fluorescence intensity measurements were obtained by subtracting the readings from 200 μl of DMEM without Zinpyr-1 loaded cells. The relative zinc levels were calculated by normalizing to the measurements of DMSO-treated samples. The results were expressed as means ± sd of three independent experiments.

### Cell proliferation assay

Cell proliferation was measured using the CellTiter 96 AQueous One Solution Cell Proliferation Assay (Promega) following manufacturer's instruction. 1 × 10^5^ MCF-7 cells per well were plated in 96-well plates. The cells in triplicates were then treated as indicated, and the plates were read on a TECAN SPARK Microplate Reader (Tecan) at a wavelength of 490 nm. The results were expressed as means ± sd of three independent experiments.

### Colony formation assay

5 × 10^4^ MCF-7 cells (on 35 mm dishes), MCF-7 with tetracycline-inducible MDMX overexpression (on 6-well plate with or without 2.25 μg/ml of tetracycline), or MCF-7 transfected with siRNA targeting TRPM7 (on 6-well plate 6 h after transfection) were plated. The cells were treated with indicated drugs for 24 h, and then fresh DMEM medium was added and replaced every other day. Five to seven days after treatment, the cells were fixed and stained with crystal violet (0.05% in 20% ethanol). The crystal violet stained cells were scanned using LI-COR Odyssey CLx imaging system. The intensity of crystal violet signal in each well was quantified to represent the cell numbers in the well.

### Wound healing assay

About 2 × 10^6^ cells MCF-7 were plated (for experiments with TRPM7 depletion, MCF-7 cells were plated 6 h after siRNA transfection) to reach ∼90% confluence, the cells were starved with DMEM plus 0.5% FBS for 24 h. Then the cells were washed twice with 1× PBS, scratched with a 200-μl pipette tip to produce a straight cell-free “wound,” and washed again with 1× PBS to remove debris followed by addition of fresh DMEM plus 0.5% FBS DMEM with DMSO or NS8593 (30 μM) as indicated. Images were taken at 0 or 24 h after scratch using a phase-contrast microscope (Nikon, Eclipse Ts2) with 10× magnification. The healing areas were measured using MRI Wound Healing Tool (http://dev.mri.cnrs.fr/projects/imagej-macros/wiki/Wound_Healing_Tool) in Image J. Three different scratched areas for each dish/well were imaged and measured. Percentage of healed wound area at 24 h related to 0 h time point was graphed as percentage of healed area = (area 0 h − area 24 h)/area 0 h × 100%.

## Data availability

All data are contained within the manuscript.

## Supporting information

This article contains supporting information.

## Conflict of interest

The authors declare that they have no conflicts of interest with the contents of this article.
