# Supplementary material for: The ion channel TRPM7 regulates zinc-depletion-induced MDMX degradation
Source: J Biol Chem. 2021 Oct 8;297(5):101292. doi: 10.1016/j.jbc.2021.101292 (PMC8561006; doi:10.1016/j.jbc.2021.101292)

The ion channel TRPM7 regulates zinc depletion-induced MDMX degradation

Herui Wang[1], Bin Li[1], Kulsum Asha[1], Ryan L. Pangilinan[1], Asha Thuraisamy[1], Harman Chopra[1], Susumu Rokudai[2], Yong Yu[1], Carol L. Prives[3], and Yan Zhu[1, #]

Supporting Information

Experimental Procedures

Cell culture and plasmids

HEK293T cells and MCF-7 cells were cultured in DMEM medium with 10% FBS (Fetal Bovine serum, Gemini Bio-products) and 1% penicillin streptomycin (Gibco) at 37°C, 5% CO_2.

To establish U2OS MDMX S367L mutant cells, the left (anti-sense; GGAAGGTTTCTGAAAGGAAGTAC) and right (sense; CAAATCCTAGGCTAGATCACTGG) sgRNAs were cloned into pX335 (Addgene #42335) to cut the genome DNA in the intron 10 of mdmx gene. Donor vector was designed to introduce mutation S367L (TCG to TTG) in exon 11 of mdmx gene with a FNF selection cassette flanked by left and right homologous arms and cloned into pFNF (Addgene #22687). The donor and CRISPR plasmids were transfected into U2OS cells, followed by selection with G418 (800 μg/ml) for 2 to 3 weeks. The resistant clones were expanded and examined for FNF insertion on the mdmx locus by PCR. The corrected clones were confirmed by both genome and cDNA sequencing. To serve as controls, isogenic wild-type clones were also obtained through the same selection process. We obtained two heterozygous (#12, S367L_1; #20, S367L_2) and one homozygous (#23, S367L_3) CRISPR cell lines. As the homozygous cell line was somewhat unstable and expressing much less MDMX, we only used the two heterozygous cell lines in this study.

Antibodies and reagents

Antibodies and reagents that were used only in supplemental data section are: phosphor-Histone H2A.X (Ser139) (SC-517348; Santa Cruz Biotechnology); HLI373 (Sigma); Compound 1 (Sigma); MD-224 (30 nM, MedChem Express); Etoposide (Sigma); and Zip7 siRNA (sc-76962; Santa Cruz Biotechnology).

ROS detection assay

2.2 $\times 10^4$ MCF-7 cells per well were plated in triplicates for 5 experimental groups on 96-well plate. Eighteen hours later, two sets of triplicate cells were treated with DMSO or TPEN (5 μM) for 8 hours and cellular ROS levels were measured using ROS Detection Cell-Based Assay Kit (DCFDA) (item 601520; Cayman Chemical) following the manufacture's procedure. 10 μM of DCFDA was used in the assay for MCF-7 cells. Positive control (pyocyanin treatment), negative control (N-actyl cysteine treatment), and no treatment control were also included for measurement in triplicates. Total DCFDA (2,7-Dichlorofluoroscin Diacetate) fluorescence was measured using an excitation wavelength at 480 nm and emission wavelength at 530 nm.

Purification of MDM2 or MDM2/MDMX Complex and mass spectrometry

HEK293T cells were transfected with Flag-MDM2 or Flag-MDM2 plus Myc-MDMX. Potential differential binding partners for MDM2 alone or MDM2/MDMX heterodimer were purified and subject to mass spectrometry analysis as previously described. Briefly, the HEK293T cells (1 x 10^10) were transfected with a Flag-MDM2 or Flag-MDM2 plus Myc-MDMX at a ratio of 10:3. Total cell lysates were prepared and subjected to immunoprecipitation with anti-FLAG monoclonal antibody (M2)-conjugated beads (Sigma-Aldrich, St. Louis, MO). The beads were washed eight times with lysis buffer and then eluted by incubating with 0.2 mg/ml FLAG peptide (Sigma-Aldrich) for 2 hrs. The eluate was concentrated and separated on a

Tris-Glycine gradient gel (4-20%). Proteins on the gel were stained with GelCode Blue (Thermo Fischer Scientific, Waltham, MA). Differentially pulled-down protein bands were excised, destained with 25 mM ammonium bicarbonate and 50% acetonitrile, dried, digested with sequence grade modified trypsin (Promega, Fitchburg, WI) in 50 mM Tris-HCl [pH 7.6], extracted with 5% trifluoroacetic acid (TFA), 50% acetonitrile, and subjected to MALDI-TOF mass spectrometry analysis. Mass spectrometry search parameters: Peaklist-generating software: Hitachi Data explorer; search Engine: MASCOT Daemon; sequence database: Swiss Prot (ver.2015.08); threshhold scores: P-value < 0.5, MSCOT Score > 10. The raw data is deposit to MassIVE, a publicly accessible repository (access number: MSV000087390).

Measurement of Intracellular Zn^{2+} with flow cytometer

MCF-7 cells were incubated with 5 µM Zinpyr-1 in DMEM for 30 min at 37°C. Then the cells were washed twice with 1×PBS, trypsinized, and resuspended. Aliquots of the cell suspension were subjected to DMSO, NS8593 (50 µM), TPEN (5 µM), or $ZnSO_4$ (5 µM) alone or in combination treatments for 30 min at 37°C. Then the cells were passed through a 40 µm cell strainer (VWR) and subjected to flow cytometer measurements for green fluorescence signals with a Guava easyCyte System (ExpressPro; EMD Millipore Corporation, Inc). The FCS Express 7 (De Novo software) was used for cytometry data analysis and visualization of histogram plots overlay.

Supplemental Figure Legends

Figure S1. TPEN treatment induces minimal ROS in MCF7 cells. TPEN treatment induces minimal ROS production. 2.2 $\times 10^4$ MCF7 cells per well were plated on 96-well plates in triplicates for 5 experimental groups. Eighteen hours later, two sets of triplicate cells were treated with DMSO or TPEN (5 µM) for 8 hours and cellular ROS levels were measured using ROS Detection Cell-Based Assay Kit (DCFDA). Positive control (pyocyanin treatment), negative control (N-actyl cysteine treatment), and no treatment control were also included in triplicates following the manufacture's procedure. Total DCFDA (2,7-Dichlorofluoroscin Diacetate) fluorescence was measured using an excitation wavelength at 480 nm and emission wavelength at 530 nm.

Figure S2. TPEN-induced MDMX degradation is independent of MDM2. (A) MDM2 inhibitors does not protect MDMX from TPEN-induced degradation. MCF-7 cells were treated with DMSO (control) and 5 µM TPEN for 5 hours and then $ZnSO_4$ (25 µM), compound 1 (Comp1: 6, 12, or 24 µM), or HLI373 (5 or 10 µM) was added as indicated. Five hours later, the cells were harvested and total cell lysates were analyzed by immunoblotting with indicated antibodies. (B) MDM2 PROTAC degrader MD-224 does not protect MDMX from TPEN-induced degradation. MCF-7 cells were pre-treated with DMSO (control) and 30 nM MD-224 for 2 hours, and then treated with DMSO (control) or 5 µM TPEN for 5 hours. After that, 25 µM $ZnSO_4$ was added back in one set of TPEN treated cells as indicated. Five hours later, the cells were harvested and total cell lysates were analyzed by immunoblotting with indicated antibodies. All the immunoblotting experiments were repeated at least twice to ensure the reproducibility of the results.

Figure S3. TPEN induces DNA damage to a much lesser extent than etoposide. MCF-7 cells were treated with DMSO, 5 µM TPEN, or 15 µM etoposide for 8 hours. In one set of the TPEN treated cells, 25 µM $ZnSO_4$ was added back 3 hours after TPEN treatment and the cells were incubated for another 5 hours. Total cell lysates were then prepared and subjected to immunoblotting with anti-γH2AX or anti-actin antibodies. All the immunoblotting experiments were repeated at least twice to ensure the reproducibility of the results.

Figure S4. TRPM7 was identified as a potential binding protein for MDM2/MDMX heterodimer. (A) Twenty plates (150 mm) of HEK293T cells were each transfected with Flag-MDM2 (10 µg) or Flag-MDM2 (10 µg) plus Myc-MDMX (3 µg). Total cell lysates were prepared and subjected to immunoprecipitation with anti-Flag antibody. The co-immunoprecipitated proteins were analyzed by SDS-PAGE followed by silver staining. Differentially pulled-down protein bands were excised from the gel and subjected to mass-spectrometry analysis. (B) A list of relative identified proteins including TRPM7 was shown. The raw data was deposit to MassIVE, a publicly accessible repository (access number: MSV000087390). (C) Mass spectrometry search parameters used for analysis were listed. (D) TRPM7 interacts with MDMX. HEK293T cells were transfected with Flag-TRPM7 (1 µg), Myc-MDMX (0.3 µg) or in combination as indicated. Twenty-four after transfection, cells were harvested and total cell lysates were prepared and subjected to immunoprecipitation with anti-Flag antibody. The co-immunoprecipitated proteins were analyzed by immunoblotting with anti-Flag or anti-MDMX antibodies. The immunoblotting experiments were repeated at least twice to ensure the reproducibility of the results.

Figure S5. mRNA levels of MDMX remain constant upon TPRM7 overexpression or inhibition. (A) TPRM7 overexpression doesn't affect mRNA levels of MDMX. HEK293T cells

were plated in 60 mm dishes and transfected with Flag-TRPM7 variants (2 µg). Twenty-four hours after transfection, cells were trypsinized and re-plated into 35 mm dishes. Twenty-four hours after re-plating, the cells were treated with DMSO, 4 µM, or 5 µM TPEN for 5 hours. Then 5 µM ZnSO4 was added into one of TPEN-treated dish. One hour later, the cells were harvested and total RNA was extracted. Quantitative RT-PCR was carried out to check mRNA levels of MDMX. (B) Inhibition of TPRM7 by siRNA knockdown doesn't affect mRNA levels of MDMX. MCF-7 cells were treated with control siRNA or siRNA targeting TRPM7 (siTRPM7-2). Forty-eight hours later, the cells were treated with 5 µM TPEN for 5 hours and then incubated with or without 5 µM $ZnSO_4$ for another 5 hours. The cells were harvested and total RNA was extracted. Quantitative RT-PCR was carried out to check mRNA levels of MDMX. (C) Inhibition of TRPM7 by NS8593 doesn't affect mRNA levels of MDMX. MCF-7 cells were treated with 5 µM TPEN for 5 hours and then 5 µM $ZnSO_4$ were added in the absence of presence of 50 µM NS8593. The cells were harvested at 5 hours after $ZnSO_4$ and NS8593 treatment. The cells were harvested and total RNA was extracted. Quantitative RT-PCR was carried out to check mRNA levels of MDMX. In all experiments, no statistical significance was observed.

Figure S6. TRPM7 modulates intracellular levels of Zn^{2+}. MCF-7 cells (A) or MCF-7 cells transfected with control siRNA or siRNA targeting TRPM7 (siTRPM7_2) (B; 50 nM each; 72 hours after transfection) were loaded with Zinpyr-1 (5 µM) for 30 min at 37°C. Then the cells were trypsinized and treated as indicated (DMSO, 5 µM TPEN, 50 µM NS8593, alone or in combination with 5 µM $ZnSO_4$) for 30 min at 37°C followed by flow-cytometry measurements as described in Experimental Procedures. The shift of green fluorescence peak in the overlayed histogram plot indicated the relative zinc level changes upon different treatment. MCF-7 cells

without Zinpyr-1 loading (Unstained) were also measured and used as cell auto-fluorescence negative control.

Figure S7. Ablation of Zip7 attenuates Zn^{2+}-mediated recovery of MDMX in the presence of TPEN. MCF7 cells were treated with control siRNA or siRNA targeting Zip7. Forty-eight hours later, cells were treated with 5 μM TPEN for 5 hours and then treated with or without 5 μM $ZnSO_4$ for another 5 hours. The cells were harvested and total cell lysates were analyzed by immunoblotting with indicated antibodies. The immunoblotting experiments were repeated at least twice to ensure the reproducibility of the results.

Wang et al Figure S1

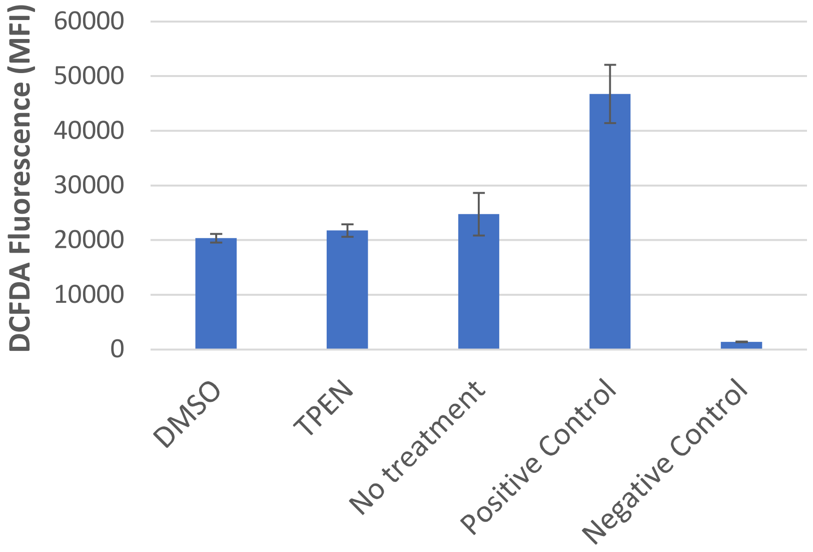

Wang et al Figure S2

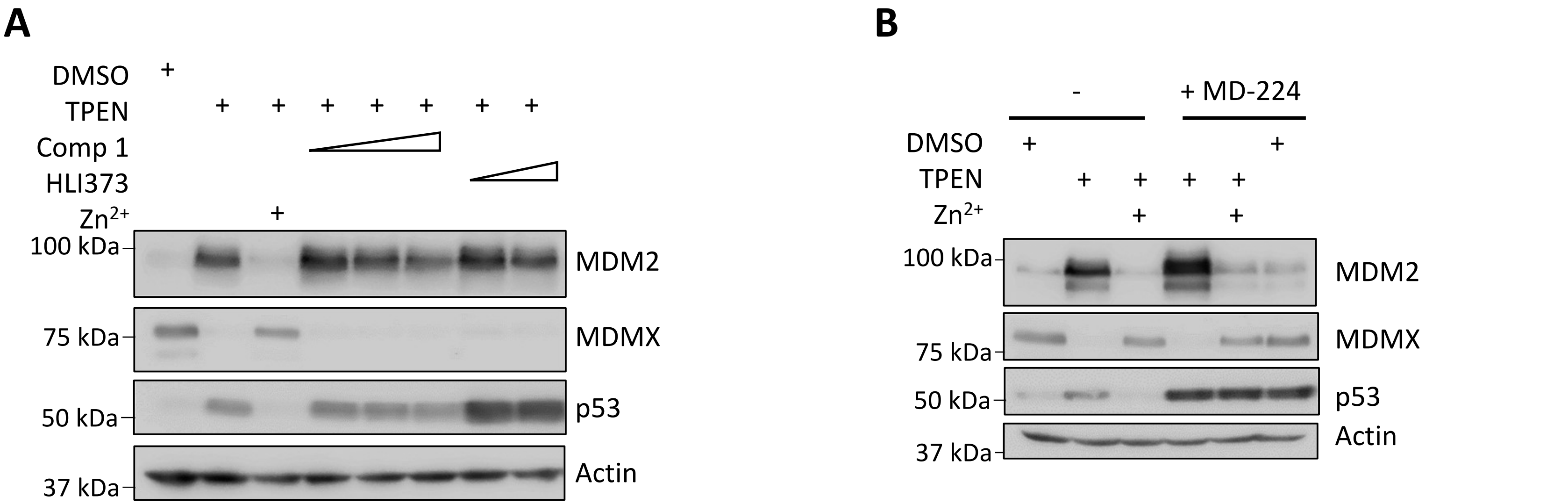

Wang et al Figure S3

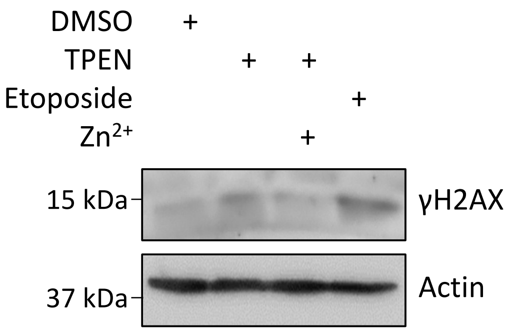

A

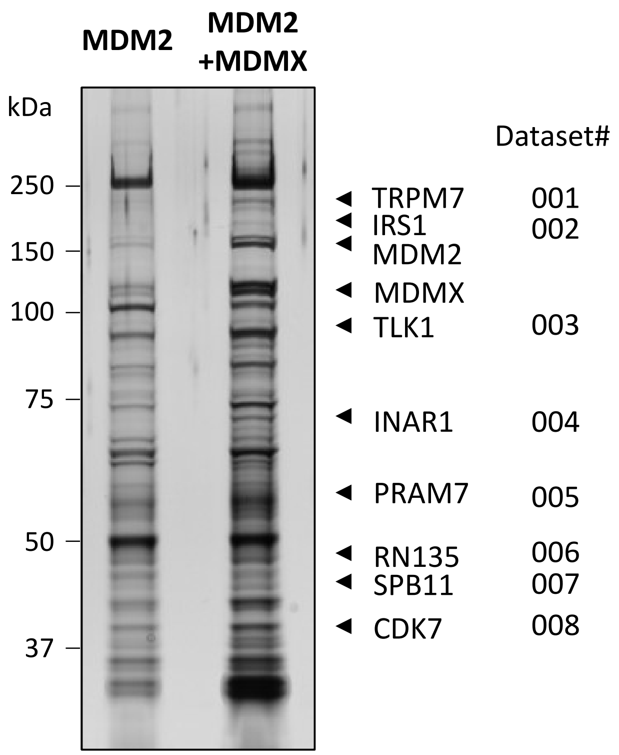

C

Mass spectrometry search parameters:

- Peaklist-generating software: Hitachi Data explorer
- Search Engine: MASCOT Daemon
- Sequence database: Swiss Prot (ver.2015.08)
- Number of entries: 8 (001~008)
- Protease used to generate peptides: Trypsine
- Fixed modification: Carbamidomethyl (C)
- Variable modification : [Ser,Thr] phosphorylation
- Tolerance for precursor ions: 0.5 Da
- Tolerance for fragment ions: 0.8 Da

D

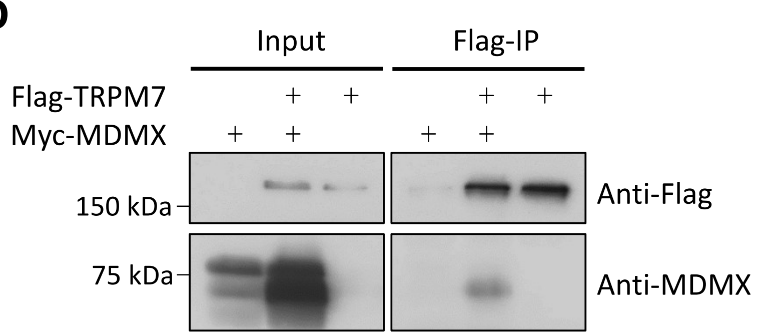

B

The list of the relative identified proteins

	Dataset	Score	Mr(expt)	Num. of Matches	emPAI	Description
TRPM7	001	18	214670	2	0.01	Transient receptor potential cation channel M7
IRS1	002	14	132706	2	0.02	Insulin receptor substrate 1
TLK1	003	15	87216	2	0.03	Ser/Thr kinase 1
INAR1	004	15	64112	2	0.05	Interferon alpha/beta receptor 1
PRAM7	005	19	54929	2	0.06	PRAME Family member 7
RN135	006	14	48827	2	0.06	E3 ubiquitin-protein ligase RNF135
SPB11	007	14	44356	3	0.07	Serpin B11
CDK7	008	13	39299	2	0.07	Cyclin-dependent kinase 7

Wang et al Figure S5

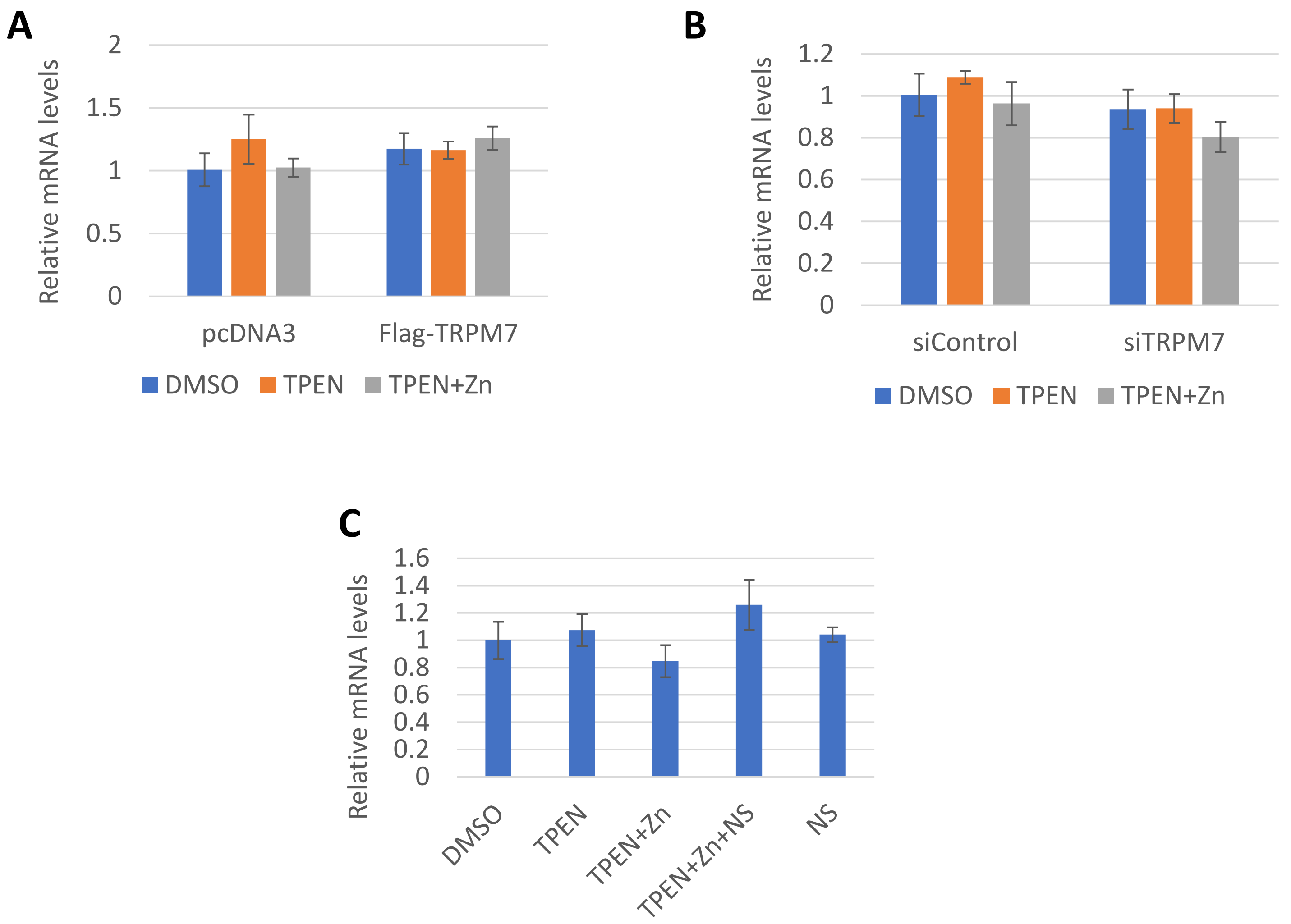

Wang et al Figure S6

A

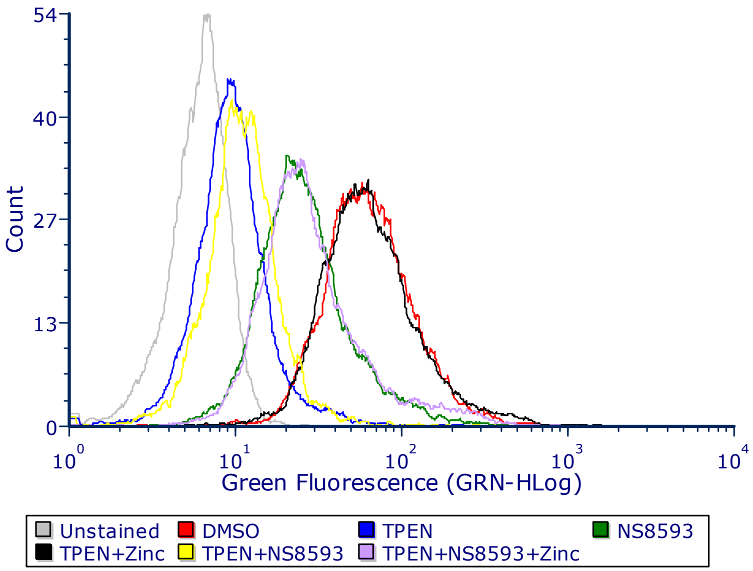

B

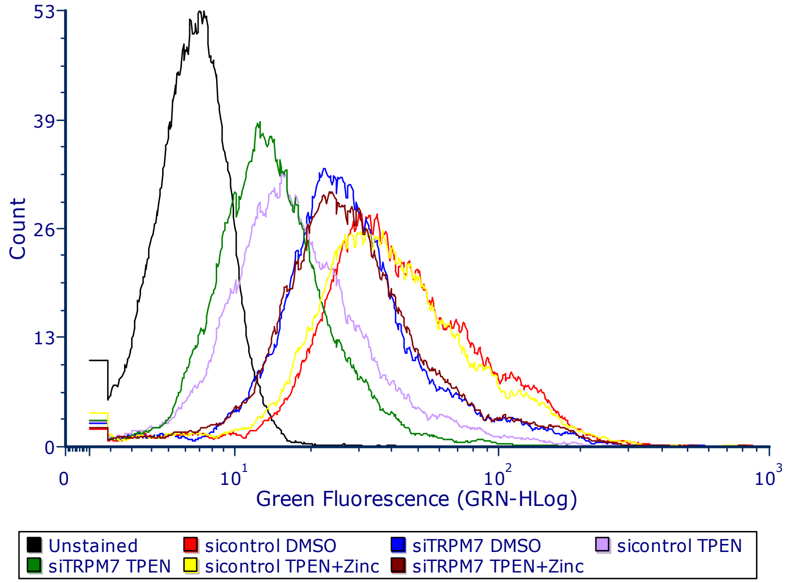

Wang et al Figure S7

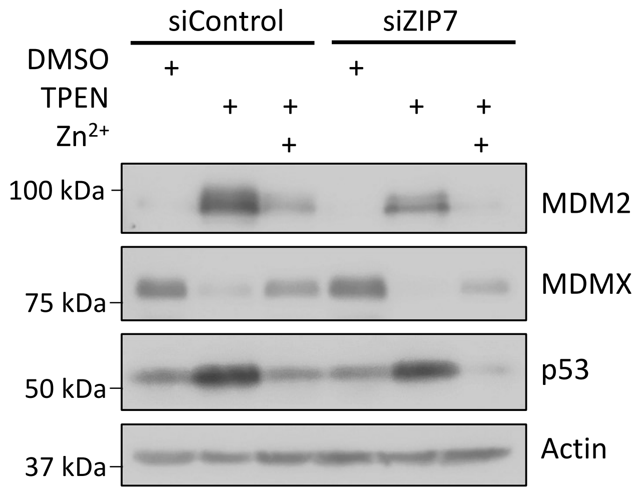

Supplement: Supporting information [file mmc1.pdf]
